# Systems engineering of engineered live biotherapeutics: A discovery-to-translation framework for streamlining microbiome therapeutic development

**DOI:** 10.1016/j.jconrel.2026.115160

**Published:** 2026-07-06

**Authors:** Noah T. Hutchinson, Zeyang Pang, Collins I. Chimezie, Brian Hamp, Amber E. Haley, Jiahe Li

**Affiliations:** a Department of Biomedical Engineering, University of Michigan, Ann Arbor, MI 48109, United States; b Cellular and Molecular Biology Program, University of Michigan, Ann Arbor, MI, 48109, United States; c University of Michigan Rogel Cancer Center, University of Michigan Medical School, Ann Arbor, MI, 48109, United States; d Program in Chemical Biology, Life Sciences Institute, University of Michigan, Ann Arbor, MI, 48109, United States

**Keywords:** Engineered live biotherapeutic products, Gut microbiome engineering, Cellular kinetics/pharmacodynamics, Design-build-test-learn, Synthetic biology, Conjugative plasmids, Engineered bacteriophages, Flux balance analysis, Gut-on-a-chip, New approach methodologies

## Abstract

Despite the vast opportunities for therapeutic manipulation of the gut microbiome, recent late-stage clinical failures of engineered live biotherapeutic products (eLBPs) highlight critical knowledge gaps in ecological barriers and community dynamics. In this review, we propose repurposing the current eLBP toolkit as a set of discovery instruments that yield quantitative outputs for predictive modeling. We examine cutting-edge approaches in microbiome engineering and outline opportunities for their use in tandem with systems engineering methodology to conduct functional probing that establishes quantitative parameters describing community resilience, metabolic flux, and host-microbe interactions. Next, in light of FDA guidance on New Approach Methodologies, we detail how *in silico* and *in vitro* modeling approaches can be combined and leveraged not only for *a priori* triage of unviable designs, but can also be integrated into design-build-test-learn (DBTL) pipelines for functional forecasting. Building off an emerging cellular kinetics/pharmacodynamics (CK/PD) framework, we develop a Bayesian updating workflow that encapsulates eLBP-adapted equivalents of pharmacological parameters such as Cmax, Tmax, and AUC. Further, we adapt this framework for adaptive or prospective use, rather than purely retrospective application, supporting trial design rather than post-hoc analysis. This approach repositions eLBP development from an empirical, intuition-based process toward a predictive, model-informed pipeline that aligns with emerging regulatory frameworks.

## 1. Introduction

The human gut harbors a vast consortium of trillions of microorganisms with a wide range of important contributions to human health and development. To synthetic biologists, bioengineers, and drug developers, this expansive community with a rich and diverse metabolite pool presents nearly endless opportunities for the development of novel therapeutics. Engineered live biotherapeutic products (eLBPs) have garnered increased attention as of late for their potential to serve as noninvasive, adaptable, and efficacious solutions capable of circumventing many prominent clinical barriers. Advances in genetic engineering and microbiology now enable programming of organisms such as commensal bacteria, probiotics, and phages to perform a range of additional functions. In the gut, these specialized systems can report on physiologic conditions, eliminate pathogens, edit microbial genes, and produce therapeutic compounds. These capabilities fall into three broad functional categories: sensing and quantifying the gut environment, perturbing and delivering targeted interventions, and controlling and modulating metabolic output. Despite the excitement and promising performance in early clinical phases, to date, no eLBP approaches have received FDA approval and entered the market as approved drugs for clinical use [1–5]. This can be blamed, in part, on the relatively limited knowledge base surrounding the governing homeostatic forces, functional contributions, and evolutionary dynamics of the host microbiota. Despite promising preclinical validation and encouraging early-phase clinical results, recent late-stage failures illustrate this gap concretely. Prominent programs such as Synlogic’s SYNB1934 for phenylketonuria (PKU) and Novome Biotechnologies’ engineered oxalate-degrading therapeutic have struggled to meet endpoints due to insufficient prediction of gut compatibility and genetic stability [5]. This trend stresses the need for quantitative frameworks that can anticipate these challenges before significant clinical investment is committed.

The structural resilience of the gut microbiota represents an acknowledged yet poorly understood paradox within the field. On one hand, these communities are highly resilient to minor insults and consistently revert to baseline after minor environmental alterations such as host dietary or behavior changes. In contrast, severe perturbations such as antibiotic use can cause lasting disruptions which require months to years for full or even partial recovery. From an ecological perspective, functional redundancies and the presence of ‘keystone species’ have been proposed to explain these characteristics, yet our mechanistic understanding remains surface level [6,7]. Compounding this challenge, a comprehensive definition of a ‘healthy’ microbiota remains largely elusive despite significant research effort, leaving many eLBP designers without defined target states. A deeper understanding of host-microbe interactions, temporal dynamics, and spatial distribution within the gut is needed. Identifying structural ‘tipping points’, and niche provision will be critical for predictable and efficacious eLBP design. To capture these dynamic complexities, a highly adaptive and analytically powerful toolkit is needed.

In this review, we summarize the current state of the art in eLBP and nanotechnologies for both therapeutic and mechanistic discovery purposes. We outline how recent advancements in conjugative plasmids, engineered phage technology, bacterial biosensors, nanoprobes, and reporter methods can be used to further unravel the unknown characteristics governing microbial ecosystems in the gut. Further, we discuss how current computational methods and in vitro screening can be paired for the detection of unviable designs *a priori*. Finally, we expand on emerging cellular kinetic-pharmacodynamic (CK/PD) models to develop a framework and roadmap for mapping quantitative pharmacokinetic parameters to meaningful outcome metrics in eLBP trials. Our goal is to provide insights that can streamline design-build-test-learn (DBTL) workflows and de-risk translational efforts.

## 2. The engineered toolkit: delivery vehicles and synthetic circuits for gut microbiome interrogation

To target this complex ecosystem, tools have emerged that can both probe and manipulate this gut ecosystem with precision. The tools described in the following section fall into two functional categories: delivery vehicles that introduce genetic cargo into specific community members (2.1), and synthetic circuits that enable quantitative sensing and programmable control of microbial behavior (2.2). Together, these platforms represent the current state of the art in microbiome engineering. Each tool provides specific mechanistic advantages that feed into the discovery experiments described in section 3, and translational frameworks in sections 4 and 5.

### 2.1. Delivery tools for precision genome editing

#### 2.1.1. Engineered phages

##### Phage backbone selection and life cycle considerations.

Engineered bacteriophages serve as programmable, species-specific delivery vehicles for complex genetic cargo. The inclusion of CRISPR nucleases, base/prime editors, and dynamic circuits enables targeted knockout, tuning, probing, or modification of bacterial functions within complex microbial communities. Phage selection must account for native host specificity and life cycle, which dictate replication strategy, cargo stability, and perturbation dynamics. Unlike bacteria, phages employ distinct replication strategies that directly shape their engineering utility. Strictly lytic phages (e.g., T7, jumbo *Pseudomonas* phages phiKZ/MIJ3/fMGyn-Pae01) kill the host upon lysis, temperate phages (e.g λ, Klebsiella NAR688) can integrate prophages or enter lytic cycles, and non-lytic phages (e.g., M13) continuously secrete phage particles without host death. The life cycle defines whether a phage functions as a pulse perturbation or a sustained modulating tool, as summarized in Table 1. From an engineering standpoint, lytic phages implement targeted pulse perturbation, eliminating a target population and allowing the community to shift towards a new equilibrium [8–12]. Lytic delivery options can have distinct environmental implications that will produce immediate population-level effects, such as killing or expression of phage resistance. Temperate phages are ideal for delivering proteins or CRISPR-Cas systems that can repress gene expression or mediate gene modifications throughout a cell’s lifetime. For example, delivering CRISPR-nCas9 Base Editors via lytic phages would not sustain repression over long periods in a population, because lytic phages either remove the target bacterial population or drive phage resistance, preventing sustained circuit function. Using lysogenic or non-lytic phages, such as λ, provides a durable means to sustain genetic modification while preserving the community at large [8,10,13,14]. By contrast, Filamentous M13 uses non-lytic delivery, maintaining phage stability for high-protein surface display, which is ideal for screening libraries and dynamic circuit deployment in F^+^ *E. coli* [15,16]. The surface of M13, composed of the pVIII subunits, supports the construction of hybrid nanoplatforms that facilitate phage display and chemical modifications targeting pathogens or specific cancer markers [17]. This bioconjugation strategy was first demonstrated for rapid colorimetric detection of bacterial species [18], and subsequently applied to controlled photothermal ablation of specific bacterial species [19]. These efforts demonstrated that chimeric phage-directed gold nanorod platforms can achieve killing without conventional lytic mechanisms. Beyond nanoplatform applications, M13 phagemids have also been used to deliver CRISPRi sgRNAs intercellularly to implement multicellular logic gates (YES, NOT, AND, AND-AND-NOT) in *E. coli* consortia, enabling distributed biocomputation via phage-mediated communication [20]. Jumbo Pseudomonas phages (e.g., phiKZ, MIJ3, fMGyn-Pae01) and Klebsiella telomere phages extend these designs by targeting multidrug-resistant (MDR) pathogens, including *Pseudomonas aeruginosa* and carbapenem-resistant *Klebsiella*. Their utility stems primarily from payload capacity: their genomes exceed 200 kb compared to T7’s ~40 kb, accommodating larger and more complex genetic circuits, including multi-enzyme systems and CRISPR-Cas13a constructs [8,13,21,22].

##### Genetic cargo selection.

Beyond phage backbone selection, the choice of genetic cargo determines the intended bacterial interaction or modification. CRISPR-Cas9 nucleases packaged in lytic phages enable strain-specific killing by targeting essential genes, making them ideal for eliminating MDR communities [8,10]. CRISPRi (dCas9) delivered via temperate phages provides a sustainable and tunable form of gene repression without DNA cleavage, supporting dose-response studies of metabolic or stress-response pathways [10,23]. Cytosine/Adenine base and prime editors introduce scarless point mutations or premature stop codons for precise allelic replacement or knockouts. Notably, both CRISPRi and base/prime editors avoid double-strand DNA breaks, preventing activation of the DNA damage response (DDR). This ensures stable prophage integration, making these modifications heritable across cell divisions while also preventing bacterial stress responses that may destabilize the community [10,23]. Phages can also be used to deliver multiplex guide RNA (mgRNA) arrays using CRISPRi or DNA-editing CRISPR-Cas transposase (DART), providing simultaneous knockout of multiple genes within a single strain. Successful genetic integration targeting essential genes thyA and lacZ has been demonstrated using these systems [24]. Using multiple gRNAs creates biochemical circuits that respond to various inputs, either repressing genes or inserting other genes that will affect metabolic pathways down the line [10,25]. Non-CRISPR cargos such as antibacterial toxins, exopolysaccharide (EPS) depolymerases, and recombinases offer alternative routes to bacterial killing or genomic knockouts/knock-ins without relying on Cas machinery [8].

##### Clinical applications.

Engineered phage platforms have demonstrated safety and direct clinical promise, with multiple cocktail approaches entering and/or surpassing phase I/II trials for pathobiont removal or ecological manipulation. Two completed trials (NCT04191148 and NCT05277350) have displayed the safety, efficacy, and specificity of orally administered phages in reducing gut abundance of *E. coli* strains associated with urinary tract infections. The PreforPro trial (NCT04511221) compared treatment with a standard probiotic (Bifidobacterium animalis subsp. lactis) to a combination approach of the probiotic with their PreforPro-prepped phage for patients with mild gastrointestinal (GI) symptoms [26]. The addition of the PreforPro-prepared phage potentiated probiotic efficacy, inducing significant improvements in GI inflammation and marginal improvements in colon pain in the treated groups. Beyond microbiome-targeted applications, M13 phage particles have been engineered with specific gene expression of imaging agents and therapeutic payloads for targeted in vivo delivery [27]. This demonstrates that precise phage architecture can directly influence tissue penetration and retention in complex biological environments, principles which are equally applicable to mucosal delivery in the gastrointestinal tract. Collectively, these platforms are not only therapeutic candidates but also powerful experimental vehicles: by combining host range, life cycle, and programmable cargo, they enable controlled perturbations of defined community members. As we discuss in later sections, these functions can be leveraged to dissect resilience, metabolism, and host responses.

##### Limitations.

Despite their precision, engineered platforms carry inherent limitations that must be weighed against their utility. Currently, phage therapy is in its early stages, and further research can improve its efficiency and address some of its drawbacks. Host range remains a fundamental constraint. While strain specificity is advantageous for targeted intervention, it limits applicability across diverse community compositions, necessitating cocktail approaches for wide coverage. Additionally, phage resistance can emerge when the multiplicity of infection (MOI) fails to eliminate the entire target population, leaving escape mutants and promoting phage-bacteria co-evolution within a community. For these specific obstacles, mitigation strategies are being actively explored. The use of phage cocktails can cover a broader range of bacterial targets, and the exploitation of ‘bacterial phage resistance’ via competing probiotics can suppress pathogen growth [28,29]. Another concern lies in the release of intracellular bacterial toxins and metabolites following phage lysis, which can transiently perturb bystander community members regardless of therapeutic intent [14,30]. Finally, large genetic payloads, including CRISPR arrays, base editor cassettes, or surface display constructs, impose a metabolic burden that constrains the complexity of circuits that can be stably maintained in vivo [16,31,32].

#### 2.1.2. Conjugative plasmids

##### Overview, subtypes, and engineering utility.

Conjugative plasmids are a critical tool that prokaryotes possess for acquiring new genetic elements and passing them along via horizontal gene transfer. These plasmids contain all the elements necessary for both self-propagation during prokaryotic reproduction (vertical transfer) and donor-recipient transmission [34,35]. Conjugative plasmids are typically much larger than other plasmids (often >100 kb) and can more easily accommodate multiple and/or bulky inserts compared to smaller plasmids or phages [36]. This property makes conjugative plasmids ideal carriers for synthetic molecular biology tools such as antibiotic resistance markers, reporter gene sequences such as fluorescent proteins, and base editors such as CRISPR-Cas9 machinery. To date, all of these tools have been used in conjunction with conjugative plasmids to analyze and modulate the gut microbiome.

Conjugative plasmids comprise a wide range of different subtypes that generally fall into one of two categories: broad-host range (BHR) or narrow-host range (NHR). BHR conjugative plasmids can spread throughout distantly related bacterial species and in some cases even cross phyla or domain boundaries. They are often responsible for adaptive responses to environmental pressures such as antibiotics, pollutants, or the presence of heavy metals or other similar toxins. At the other end of the spectrum, NHR conjugative plasmids can only transfer to and/or persist in closely related bacterial species [37,38].

Conjugative plasmids are also increasingly being utilized to test base-editing efficacy in the gut microbiome. These systems combine CRISPR-Cas9-derived machinery with the delivery system of conjugation to target pathogenic or otherwise harmful bacteria for killing. A recent murine study showed the oral delivery of conjugative plasmids containing CRISPR-Cas9 nuclease modules via non-pathogenic *E. coli* was able to significantly reduce the burden of the pathogenic *E. coli, S. enterica*, and antibiotic resistance genes in the small intestine and colon [39]. These data demonstrate how conjugative plasmid platforms can facilitate pathogen removal through oral delivery of commensal bacteria, circumventing the need for antibiotics or fecal microbiota transplant.

##### Emerging therapeutic applications.

Although conjugative plasmids exist naturally in bacterial cells, there have been several recent efforts to engineer their genetic machinery and utilize them for therapeutic or other targeted uses. Ronda *et al*. developed the Metagenomic Alteration of Gut Microbiome by In situ Conjugation (MAGIC) system in which they produced both replicative and integrative versions of conjugative plasmids. These plasmids contained both fluorescence- and antibiotic-based reporter genes. The replicative plasmids persist in the conjugated cells in plasmid form while the integrative elements transfer into the host cell’s genome. *In vitro* and *in vivo* mouse experiments showed the ability of these conjugative plasmids to alter the collective metagenome of the gut microbiome, demonstrating *in situ* microbiome editing of a large variety of established strains in their native environment [40]. More recently, Kaduwal *et al*. developed a CRISPR-Cas9 base-editor system employed within a conjugative plasmid that could effectively suppress bacterial antibiotic resistance genes. They also developed a complementary system that could reverse this suppression [41]. This study exemplifies the ability of conjugative plasmids to specifically target certain genetic sequences when used in conjunction with base editing technology. To address concerns of runaway conjugation with deleterious downstream effects, Jaafar *et al*. developed inducible conjugation systems that can be controlled by the presence of arabinose [42]. Most recently, Gelsinger et al. from the same group developed MetaEdit, which uses CRISPR-associated transposases delivered via mobile genetic elements to directly edit the genomes of native gut bacteria *in vivo*. This enables the insertion of new metabolic traits that can be selectively enriched through dietary consumption of inulin [43]. Importantly, this method showed the capability to edit previously intractable species. These developments trace a rapid progression from reporter delivery to functional *in situ* genome editing of established gut residents, illustrating the expanding capability of mobile genetic elements as tools for both therapeutic intervention and mechanistic discovery.

##### Limitations.

One of the major limitations of conjugative plasmids is the trade-off between their stability and their quantity. Conjugative plasmids typically have low copy numbers to minimize metabolic burden on the host cell, so the effectiveness and yield of the “packaged” machinery may be reduced [44]. In particular, BHR conjugative plasmids typically exist at a low copy number in host cells as part of their “stealth” strategy for transfer and reproduction. Despite this strategy, there is no guarantee that the BHR conjugative plasmid will be maintained long-term in the new host cell. Loss of unstable or metabolically costly conjugative plasmids has been observed in both environmental and clinical bacteria strains. In one study, less than 1% of conjugated host cells retained the plasmid after 35 days, highlighting the potential difficulties of using conjugative plasmids for long-term microbiome modulation and analysis. The host cells that can maintain the plasmid frequently develop compensatory adaptations to accommodate the fitness cost [45,46]. NHR conjugative plasmids commonly contain the psiB gene to inhibit the host cell’s SOS response, the so-called “manipulative” strategy of conjugation [37]. NHR plasmids’ suppression of the SOS response may enable better stability. This paradigm shows the evolutionary trade-offs at play: compared to BHR conjugative plasmids, a NHR conjugative plasmid that is better adapted to its host can ultimately be more successful in long-term persistence [45]. This trade-off should be considered when choosing a conjugative plasmid for microbiome analysis and deciding whether short-term, broad dissemination or long-term, host-specific transmission is preferred.

#### 2.1.3. Nanotechnology-based delivery systems

##### Nanotechnology for durable and precise gut delivery.

Unlike controlled laboratory conditions, the gastrointestinal tract is a highly hostile environment for oral delivery. It imposes overlapping biological, chemical, and physical barriers [47]. Proteolytic, lipolytic, and nucleolytic enzymes—including pepsin, trypsin, pancreatic lipases, and nucleases—rapidly degrade administered biomolecules, threatening the stability of gene-editing systems [48]. In parallel, the immune system can quickly recognize and clear foreign delivery tools, similar to its response to viral particles [49,50]. In parallel, physicochemical conditions further complicate delivery. Steep pH gradients and high ionic strength can destabilize carriers and promote aggregation [51,52]. Continuous mucus turnover, efflux mechanisms, and tight epithelial barriers also reduce residence time and limit payload penetration [53]. As a result, delivery efficiencies in biomolecule based approaches often remain below 10% [54].

Nanotechnology-based systems offer a complementary solution by introducing a physicochemical control layer that does not depend on microbial viability. These systems can enhance the resistance to extreme pH, bile salts, enzymatic degradation, and mechanical stress [55]. Their tunable properties enable strain- or niche-specific targeting without extensive genetic modification [56]. In addition, they support controlled cargo release and can incorporate sensing functions [57,58].

Collectively, nanomaterials function not only as carriers but as engineered platforms. They improve robustness, enhance targeting precision, and increase the translational reliability of in situ genome editing in the gut.

##### Nanotechnology in the delivery of gene editing systems.

Although *in vivo* demonstrations remain limited, CRISPR-based strategies are emerging as a promising approach for gut microbiome engineering [59]. Accordingly, current nanomaterial-enabled delivery systems are largely designed to support CRISPR-based tools and related nucleic acid cargos. These systems can be broadly classified into organic, inorganic, and hybrid platforms, each with distinct advantages.

Organic nanomaterials, particularly lipid nanoparticles (LNPs), offer strong biocompatibility and efficient nucleic acid encapsulation. For example, ~130 nm LNPs have been used to deliver CRISPR-Cas13a to suppress antibiotic resistance across multiple gut bacterial species without permanent genomic modification [43,60].

Inorganic nanomaterials, including carbon-based and metal nanoparticles, provide enhanced physicochemical stability under harsh conditions. Nitrogen-doped carbon dots have achieved efficient (>40%) CRISPR-Cas9-mediated editing of resistance genes in *E. coli*, while also enabling real-time tracking [61].

Hybrid systems combine inorganic scaffolds with functional organic components to improve stability, targeting, and release control. Representative examples include magnetite/silver core–shell nanoparticles with pH-responsive coatings for CRISPR-Cas9 delivery [62], as well as single-walled carbon nanotubes functionalized with polyethylenimine (SWCNT–PEI) composites for eliminating colistin resistance and reducing horizontal gene transfer [63].

Although this field is still at an early stage [64], these three material paradigms collectively point toward more robust, controllable, and system-oriented strategies for microbial genome engineering.

##### Limitations and integration challenges.

Despite their promise, nanomaterial-based delivery systems for gut microbial gene editing face several limitations that hinder translation. Batch-to-batch variability is a major concern. Small differences in synthesis can alter particle size, surface charge, cargo loading, and release kinetics [65]. This problem reduces reproducibility and complicates quantitative control of CRISPR/Cas efficacy [66]. In addition, fabrication and surface functionalization processes are often complex and multi-step, further increasing manufacturing difficulty. These factors limit scalability, raise costs, and complicate quality control under good manufacturing practice (GMP) standards, ultimately slowing clinical translation [67,68]. Similar translational challenges have been observed in clinically evaluated nanoparticle-based nucleic acid delivery systems. For example, CALAA-01, the first targeted siRNA nanoparticle to demonstrate RNA interference in humans, highlighted the difficulties associated with nanoparticle formulation, reproducibility, and successful clinical translation despite promising early results [69,70].

Regulatory uncertainty presents another major barrier. These platforms fall into a gray zone between biologics, gene therapies, and medical devices. This complicates classification, safety evaluation, and regulatory approval, particularly when combined with programmable systems such as CRISPR [71–73].

Taken together, these challenges highlight the need for tighter integration of nanotechnology-based delivery platforms with ecological modeling and system-level validation strategies, as discussed in subsequent sections [64].

Table 2 summarizes and compares delivery efficiency, targeting capability, loading capacity, and biosafety considerations for the three delivery platforms described in this section: engineered phages, conjugative plasmids, and nanoparticle carriers.

### 2.2. Synthetic circuitry for quantitative probing and control

#### 2.2.1. Dynamic biosensors

##### Sensing architectures and gut inflammation detection.

Synthetic biology enables the engineering of living microbes to detect diverse molecular and environmental cues, including metabolites, virulence factors, light, temperature, and radiation, and to convert these signals into diagnostic or therapeutic outputs [78,79]. Engineered microbes that interpret host-derived or environmental stimuli and couple them to internal genetic pathways can function as reporters or adaptive therapeutic agents, as discussed in section 2.2.2 [79–81]. These systems generally follow a modular architecture comprising a sensing module, a processing module, and an output module, often built upon native bacterial one-component systems (OCSs) or two-component systems (TCSs) that evolved to detect intracellular metabolites or extracellular cues [79–85].

Several variations of these systems have been designed for the detection of gut inflammation [80]. For example, a thiosulfate-responsive TCS from *Shewanella halifaxensis* was engineered into *E. coli* Nissle 1917 (EcN) to report elevated thiosulfate levels during Dextran sulfate sodium (DSS)-induced colitis. This functionality enabled in situ detection of inflammation in mice by measuring superfolder green fluorescent protein (*sf*GFP) fluorescence via flow cytometry of colon contents or fecal samples [80]. Similar sensing architectures have been coupled to therapeutic outputs, including extracellular adenosine triphosphate (eATP) degradation and chemotactic responses to nitric oxide [86–91]. One specific thiosulfate detecting system combines fluorescent reporting with integration of a base editing recorder that permanently stores the stimulus in the genome, called a memory switch [92]. Together, these platforms demonstrate how microbial biosensors can translate complex host-derived inflammatory cues into quantifiable readouts and/or therapeutic responses.

##### Sensors for spatial tracking.

In addition to genetic memory and visual cues, synthetic biology has also produced tools for spatial tracking of engineered microbes within the gut [93]. Acoustic reporter gene (ARG) systems have been used to enable non-invasive ultrasound imaging by heterologously expressing gas vesicle gene clusters. In one such example, Bourdeau et al. engineered EcN to produce gas vesicle forming proteins from *Bacillus megaterium* [93]. These intracellular gas vesicles scatter ultrasound, allowing real-time visualization of engineered bacteria and monitoring microbial localization, persistence, and biocontainment *in vivo* [93]. Additionally, Carreño et al. developed an engineered whole-cell bacterial biosensor that colonized the gut and reported metabolite availability *in situ* [94]. By coupling a sialic-acid–responsive circuit to advanced imaging approaches, biosensor activity was visualized across intestinal tissues, enabling spatially resolved monitoring of microbial metabolite sensing within the host environment.

Gold nanoparticle based approaches also have the potential to expand the landscape of dynamic microbial sensing by providing rapid, phenotypic readouts. Peng et al.’s chimeric phage-nanoparticle system directly couples bacterial recognition of *P. aeruginosa*, *V. cholerae*, and *E. coli* to an immediate optical signal. Unlike genetic circuits that require transcriptional activation or protein accumulation, this system exploited the inherent specificity and robustness of bacteriophage–host interactions to generate instant colorimetric outputs via thiol-mediated nanoparticle aggregation. This system has impressive sensitivity, with a detection limit of ~100 colony-forming units (CFU) [95]. While not yet used for gut-derived samples, the study validated the assay in complex biological matrices, including milk, urine, and swabs from an *ex vivo* porcine lung biofilm model. Together, they complement genetic memory devices, metabolite-responsive circuits, and acoustic reporter genes by providing a non-genetic, amplification-free modality for real-time pathogen detection and physiological state assessment, thus broadening the toolkit for dynamic biosensing in environments where speed, robustness, and minimal engineering overhead are essential.

##### Genetic applications.

Additionally, emerging technologies extend microbial biosensing and fluorescent reporting to genetic diagnostics. *Acinetobacter baylyi*, which naturally takes up extracellular DNA, was engineered to detect specific Kirsten rat sarcoma virus oncogene homolog (KRAS) mutations. By integrating mutation-specific recombination cassettes that activate GFP or selectable markers upon homologous recombination, this platform enables *in vivo* detection of colorectal cancer-associated somatic mutations within the gut lumen [96]. Similar approaches in *Bacillus subtilis* have demonstrated the feasibility of mutation-specific detection in complex environments [97]. While these systems highlight the potential for non-invasive, mutation-targeted diagnostics, their translational use will require improved biocontainment and non-antibiotic output modules [96,97]. Nonetheless, they represent an important emerging frontier in leveraging engineered microbes for precision gastrointestinal diagnostics.

#### 2.2.2. Programmable switches and logic gates

##### Logic processing, therapeutic responses, and in vivo memory.

Synthetic genetic circuits enable microbes to not only detect host- or environment-derived signals but also to process them through engineered logic to produce defined outputs [81,98]. These processing modules extend beyond simple ligand sensing to include memory, logic gating, and multi-input integration, allowing cells to convert continuous inputs into either graded analog responses or discrete digital states [81,83,84,96,99,100]. Through architectures such as toggle switches, integrase-based memory, and multi-input logic systems, engineered bacteria can perform decision-making, store transient events, and execute programmed therapeutic actions *in situ*.

As mentioned in the previous section, many inflammation-oriented detection models also contain response circuitry that converts host cues into actionable responses. A recent pioneering effort incorporated digital memory *in vivo* in a tetrathionate-responsive toggle switch built in the gut-native *E. coli* strain NGF-1 [101]. This system couples the TrtS/TrtR two-component sensor to a cI/Cro mutual-repression toggle, enabling transient exposure to tetrathionate, an inflammation-associated metabolite, to flip the circuit into a stable ON state. Activation induces a β-galactosidase reporter, producing blue colonies when recovered from mouse fecal samples. In colitis models, this ON state persisted for months, demonstrating durable memory of transient inflammatory episodes. Although the system lacks a second input to reset the switch, it illustrates how integrating a native inflammation sensor with a bistable toggle enables long-term recording of gut inflammatory events [81,98,101]. Additionally, several other inflammation-detection models have encoded for responsive production of therapeutic compounds such as apyrase, pyocin, and lysin, and to guide chemotactic movement in a nitric-oxide guided manner [86–88,91]. Similar sense- and-respond logic underlies “seek-and-kill” systems in which engineered *E. coli* detect pathogen-specific quorum-sensing molecules and respond by producing targeted antimicrobials to eliminate *Pseudomonas aeruginosa in vivo* [89,90].

Perhaps the most comprehensive edition, an intelligent probiotic *EcN* strain, termed i-ROBOT (intelligent Responsive Bacteria for Diagnosis and Therapy), can sense, diagnose, and treat inflammatory bowel disease (IBD) [92]. The system integrates real-time sensing and reporting, a recorder that encodes inheritable memory via base editing, and a dose-responsive therapeutic secretion module [92]. Upon detecting thiosulfate, the circuit activates a base editing (BE) recorder that permanently stores the stimulus in the genome (memory switch). In parallel, the strain produces *sf*GFP as a fluorescent reporter and secretes the immunomodulatory protein AvCystatin (*Acanthocheilonema viteae*) in a graded, dose-dependent manner proportional to thiosulfate levels *in situ*. Importantly, this therapeutic output is orthogonal to bacterial growth. However, the inducible circuit faces several limitations. First, transient and suboptimal colonization efficiency of *EcN* in the gut necessitates frequent administration Second, it does not yet have biocontainment functionality to prevent environmental escape or horizontal gene transfer. Finally, circuit stability constraints may arise from reliance on low-copy plasmids rather than chromosomal integration.

##### Non E. coli-based systems.

**Fig. 1.**
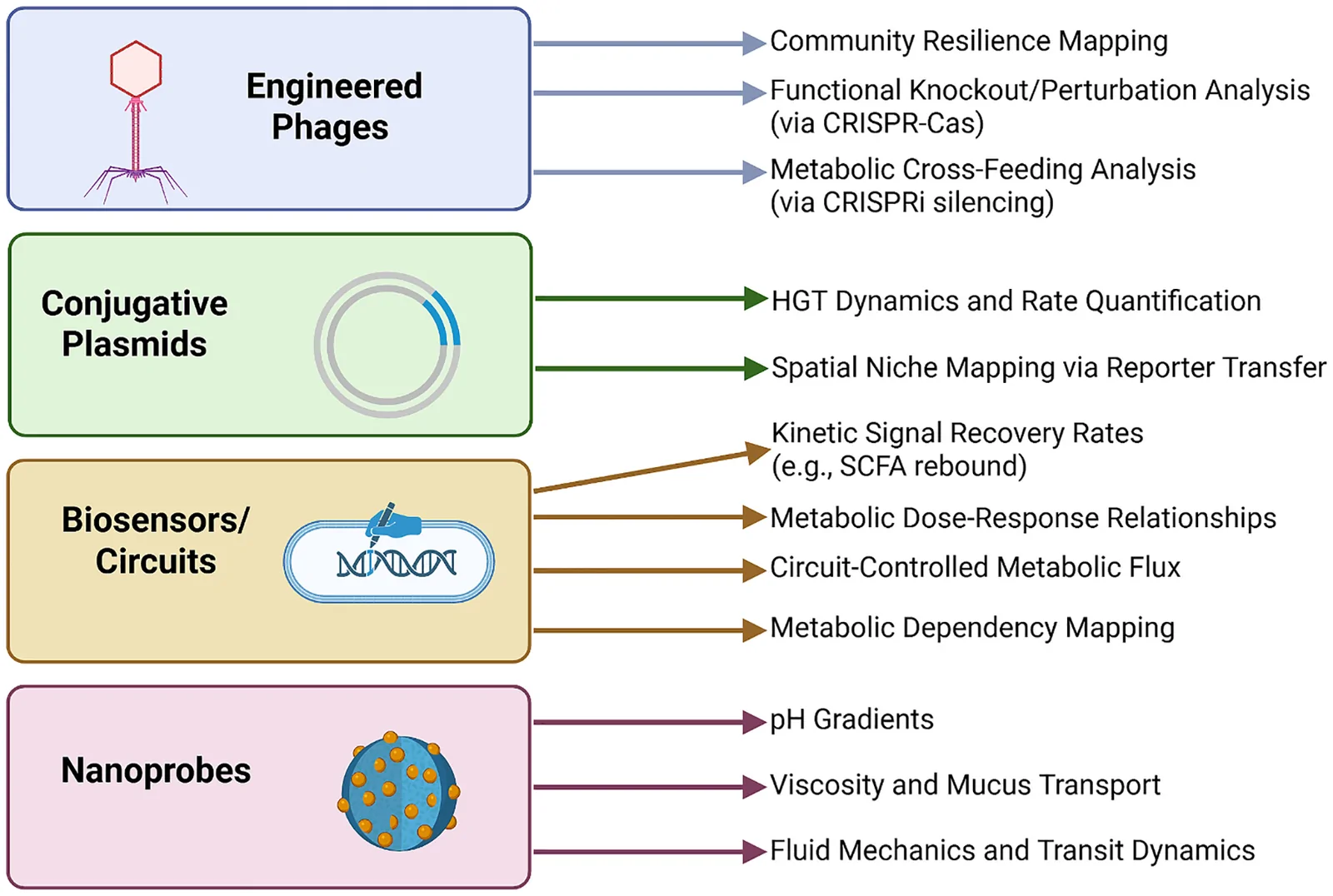
Discovery toolkit for mechanistic interrogation of the gut microbiome. Engineered phages, conjugative plasmids, biosensors/synthetic circuits, and nanoprobes each enable distinct categories of mechanistic output. Phages provide community resilience mapping through selective knockout and functional perturbation via CRISPR-Cas systems. Conjugative plasmids enable quantification of horizontal gene transfer dynamics and spatial niche mapping via reporter transfer to native gut residents. Biosensors and synthetic circuits quantify kinetic signal recovery rates, metabolic dose-response relationships, and circuit-controlled metabolic flux. Nanoprobes measure physical and mechanical parameters including pH gradients, mucus viscosity, and fluid transit dynamics. These tools collectively generate the quantitative readouts needed to parameterize the computational and translational frameworks described in Sections 4 and 5.

While most existing technologies in this space have used highly tractable *E. coli* chassis, multi-input processing has also recently been implemented in the gut commensal *Bacteroides thetaiotaomicron* (*B. theta*). *B. theta* is particularly useful for certain cases due to its ability to naturally persists at high abundance within intestinal crypts [99]. An osmolality-responsive inverter circuit was constructed by linking the opuA osmosensor to TetR, which represses downstream GFP expression. Elevated luminal osmolality activates *opuA*, increases *TetR*, and reduces GFP, yielding a tunable inverse output representing intestinal malabsorption [99]. When mice were administered graded concentrations of polyethylene glycol (PEG), the circuit produced dose-dependent, reversible reporter responses that quantitatively tracked physiologically relevant osmolality ranges. Although GFP maturation limits in vivo dynamic range under anaerobic conditions, this design demonstrates how *B. theta* can be leveraged to advance the existing microbial biosensor toolkit by supporting multi-input, quantitative, and reversible sensing architectures for long-term gut monitoring [99]. Beyond inflammation and osmolality sensing, similar inducible architectures have been applied to metabolic flux control and pathogen suppression, providing valuable tools for mechanistic discovery [102,103]. Collectively, these systems provide the quantitative, time-resolved control needed for the mechanistic discovery experiments described in section 3 (Fig. 1.).

### 2.3. Stability and safety considerations for the engineered toolkit

Across these tool categories, several safety and stability challenges must be considered prior to clinical translation. First, engineered strains and their genetic payloads are subject to evolutionary pressures in the gut environment that may encourage fitness-conferring mutations. Engineered circuit functions can be metabolically costly, thus silencing of these circuits may confer fitness advantages, gradually eroding therapeutic function across cell generations [104]. This phenomenon is well documented in plasmid-based synthetic circuits [105,106], and metabolic burden-driven selection pressure is expected to apply regardless of genomic context. Second, immunogenicity risks may arise from many engineered bacterial components such as surface-displayed proteins, foreign gene products, or novel metabolic outputs. While host adaptive immune responses to bacterial antigens is a well-established concept, the degree to which they will limit colonization, accelerate clearance, or generate off-target inflammation and present safety risks is incompletely characterized for most eLBP platforms [107,108]. Third, biocontainment remains a major barrier and active frontier in the field. Engineered fail-safe mechanisms like kill switches, which couple bacterial survival to the presence or absence of specific exogenous signals, represent an active frontier but remain in development. Processes such as horizontal gene transfer, loss of auxotrophic kill switches under metabolic rescue conditions, and phage mediated spread of engineered cargo represent distinct escape mechanisms requiring orthogonal biocontainment strategies. Comprehensive reviews of biocontainment strategies including kill switch designs and auxotrophic containment have been published elsewhere [107,109–111]. Characterizing and mitigating these failure modes requires the kind of controlled experimental approaches outlined in section 3, which aim to generate empirical data on *in vivo* circuit stability, immune interactions, and escape dynamics following perturbation.

## 3. Systems engineering for mechanistic discovery: using existing tools to unravel gut microbial dynamics

The engineered toolkit described in section II has not only therapeutic potential, but can also serve as a set of experimental levers for interrogating gut ecosystem behavior. In section 3, we outline how specific phage, plasmid, biosensor, and nanoprobe designs can be deployed in controlled perturbation studies that define community resilience (3.1) and the metabolic dynamics that shape host outcomes (3.2), visualized in Fig. 1. These experiments can generate measurable quantities that map onto the CK/PD and modeling parameters developed in sections 4 and 5, such as recovery half-life, cross-feeding coefficients, conjugation rates, and transport parameters.

### 3.1. Unraveling homeostatic forces and community resilience

#### 3.1.1. Phage-mediated functional knockouts and perturbation analysis

##### Phage knockouts for keystone identification.

Phage-mediated functional knockouts can reveal which specific community members drive ecological stability. By selectively eliminating individual strains (e.g., bacteriocin producers, nutrient scavengers), these perturbations generate quantitative data on each member’s contribution to community resilience: does the community return to its prior state, shift to a new equilibrium, or collapse [10,31,33]? Empirical frameworks for measuring these outcomes are emerging; bacteriophage-mediated perturbation of defined gut bacterial communities in chemostat bioreactors has demonstrated detectable multi-phyla compositional shifts, validating in vitro platforms for studying phage-bacteria dynamics [112]. Multiplicity of Infection (MOI) titrations are a standard dosing framework in phage therapy. MOI is defined as the ratio of phage particles to bacterial cells, and, under the Poisson infection model, can be used to estimate the theoretical fraction of bacteria that escape infection. This provides a practical quantitative basis for dosing decisions rather than a direct mechanistic measurement of killing efficiency [74]. For example, an MOI of 0.1 is expected to eliminate the most dominant cells while preserving 90% of the population uninfected; an MOI of 1 yields about 37% survival; and an MOI ≤ 5 drives nearly complete clearance. By titrating MOI from 0.1 to 10 against biofilm-producing *P. aeruginosa*, researchers can quantitatively map the ‘tipping’ point that affects community structure. This would link MOI to changes in species composition, and thereby estimate thresholds for targeting dominant vs persistent subpopulations [21,22,74].

##### Knockout study design considerations and confounds.

Selecting the right knockout target requires mechanistic hypotheses about which community members drive community stability. Metabolic cross-feeders are one high-value target class, as they supply the community with essential metabolites whose removal can restructure the entire network. However, a key confound when using lytic phages is that cell lysis releases intracellular contents, including metabolites such as acetate, which can transiently stimulate bystander growth regardless of whether the target was actively cross-feeding [32,113]. Ex vivo colon models have demonstrated that phage cocktails can eliminate target strains while preserving dominant community structure, with region-specific metabolic consequences including shifts in butyric acid production [114]. This creates misinterpretation risks by making it difficult to distinguish a strain’s metabolic contribution from the physical act of killing it [115,116]. For example, in synthetic-cross-feeding communities where *E.coli* and *S. enterica* exchange essential metabolites, T7 phage attacks on *E. coli* have resulted in *S. enterica* reaching higher concentrations compared to no-phage populations. In contrast, p22vir phage attacks on *S. enterica* have resulted in little to no change in the non-host species. Changes in these species’ populations are not because of a loss of cross-feeding dependencies but because of the release of cell debris or primary metabolites that can fuel bystander growth [117]. In an intestinal therapeutic context, an analogous lysis artifact could lead researchers to falsely classify butyrate-producing strains as a keystone cross-feeder driving community stability, when the observed bystander bloom was driven entirely by intracellular metabolite release rather than active cross-feeding. A cleaner approach is to deliver CRISPRi or a base editor via temperate phages, silencing cross-feeder genes without lysing cells [10,33]. This separates the organism’s metabolic contribution from cell death, enabling clean metabolic readouts that accurately reflect community-level dependencies. Pairing this silencing strategy with targeted Short Chain Fatty Acid (SCFA) profiling before and after gene silencing could generate quantitative data identifying which members contribute through essential metabolic nodes versus those that are functionally dispensable, directly informing which targets would destabilize community structure. The capacity of temperate phage delivery to sustain stable circuit function over time is further illustrated by M13 phagemids delivering orthogonal sgRNAs between *E. coli* cells to implement multicellular logic gates (AND-AND-NOT), quantifying how communication kinetics and resource competition shape consortium function [20]. For engineers designing live biotherapeutics, metabolomic profiling is therefore essential to avoid cascading community-wide effects from poorly chosen perturbation targets. A recurring challenge in phage-mediated perturbation is overcoming conserved bacterial stress responses that reduce phage efficacy. For example*, P. aeruginosa* activates PQS signaling during infection, coordinating community-wide phage resistance through rhamnolipid secretion. However, multiplexed gRNA circuits that can partially disrupt this dynamic have been developed, displaying the potential of sequential or two-step phage delivery strategies [30,118]. Ultimately, the value of these systems engineering approaches lies in their ability to move beyond correlation and use controlled phage-mediated perturbations to establish causal links between individual community members and the emergent properties that define microbiome health and resilience.

#### 3.1.2. Conjugative plasmid dynamics for gene transfer analysis

##### In vivo gene transfer dynamics: lessons from existing systems.

Bacteria, including enteric strains in the microbiome, already naturally use conjugative plasmids as a form of genetic evolution and horizontal gene transfer. There has thus been much interest in using engineered and synthetic conjugative plasmids with different reporter circuits to study the dynamics of bacterial interactions in the gut. For example, in the MAGIC system previously discussed, the authors designed a suite of conjugative plasmids with differing replication origins that spanned a spectrum of broad to narrow host ranges. They simultaneously performed FACS to screen for GFP^+^ bacteria while isolating transconjugants via selective antibiotic resistance (AbR) markers. After identifying the strains via 16S sequencing, they identified 19 genera across 4 phyla in total across their *in vivo* experiments. This validated their ability to transfer genetic reporters to a broad swath of the mouse gut population. Critically, their *in vitro* conjugation experiments could only successfully isolate 7 distinct genera of transconjugants via selective plating, despite similar levels of transconjugant diversity as measured by FACS metagenomics. This growth discrepancy could possibly be explained by cell density, nutrient availability, and/or the physiologically protective conditions inside the gut, underscoring the importance of investigating conjugation *in vivo* and *in situ*.

Two other key results from this study can inform future investigations into conjugation and colonization in the microbiome. Although the original donor bacteria were *E. coli* strains, conjugated *E. fergusonii* strains could similarly distribute the GFP-AbR cassette to recipients. This signals that secondary conjugation could extend the long-term stability of reporter genes. Additionally, gut-adapted donor bacteria (as opposed to the original engineered *E. coli*) were shown to be much more persistent in the gut and similarly extended the long-term stability of the conjugative payload, albeit to a narrower host range. Collectively, these results show a tradeoff occurring between the breadth and efficiency of a conjugative donor versus its persistence in the gut. Depending on the investigation in question, these parameters can be adjusted in the donor cell to achieve the desired outcome [40]. Building on this foundation, MetaEdit demonstrated that CRISPR-associated transposases delivered via mobile genetic elements can insert functional metabolic traits directly into native gut commensals *in vivo*. This includes previously intractable species, with dietary selection enabling enrichment of edited populations [43]. This capability enables perturbation experiments which add or remove a specific metabolic function from an organism within its native niche, circumventing colonization constraints associated with exogenous chassis.

##### Inducible conjugation for biomarker and Niche mapping.

Inducible conjugative plasmids have the potential to explore two related aspects of the gut microbiome, 1) rate of HGT/temporal control, and 2) presence/absence experiments to screen for nutrients and biomarkers. As noted above, in the absence of inducers (mainly small molecules such as sugars or pheromones), conjugative transfer between cells can be limited or fully suppressed depending on the basal rate of the promoter. These inducible systems exist naturally but can also be engineered into preexisting conjugative plasmids [42,76]. Relatedly, research has shown that environmental conditions can affect the rate of HGT from conjugation [119]. These discoveries can be combined to design experiments in which conjugative plasmids are designed with inducers that are triggered by specific nutrients or biomarkers of interest in the gut. The presence of conjugation (as indicated by reporter genes such as antibiotic resistance (AbR) markers or fluorescent proteins) and its rate compared to basal and/or induced states (as determined by quantitative measurements including FACS enrichment and selective CFU growth) would illuminate environmental conditions in the gut. Differential recipient analysis (i.e. 16S metagenomics) could also show which gut strains live in the presence of these biomarkers. Alternatively, postmortem assessments of colon Swiss rolls could be analyzed for transconjugants as an indicator of niche occupancy by gut microbes. With a combination of various host-range donor cells, inducers (e.g. known biomarkers of interest), and reporter genes, the absence or presence of transconjugants–and their spatial extent–could show 1) the physical locations of specific strains within the gut, 2) which nutrients are available there, and 3) of these locations and nutrients, which bacteria species are conjugating the most (i.e. conjugative “hotspots”) [120].

#### 3.1.3. Kinetic biosensing of regulatory signals

##### Biosensor platforms for longitudinal gut monitoring.

The engineered microbial biosensors described in Section 2.2.1 have utility beyond therapeutic applications: they can be deployed in longitudinal perturbation experiments, tracking fluctuations of key metabolites and signaling molecules over time with quantifiable genetic or fluorescent outputs. This capability forms the basis of kinetic biosensing, in which biosensors are used not only to detect regulatory signals but also to quantify their temporal dynamics during microbiome disruption and recovery.

Several platforms illustrate this potential. Engineered strains that detect inflammation-associated metabolites such as nitrate, tetrathionate, thiosulfate, or heme from lysed red blood cells can perform longitudinal monitoring of gut inflammation through non-invasive fecal sampling [80,121–123]. Additionally, complementary technologies containing acoustic reporting genes or image-based tracking enable detection with spatial resolution in defined gut regions [93,94]. Beyond purely microbe-based systems, hybrid device-microbe platforms have been developed that enable wireless, real-time biosensor readouts. Mimee et al.’s ingestible electronic capsule contains engineered bacteria that detect heme and trigger bioluminescence detection electronics that send photocurrent to a receiver [122]. While these approaches represent exciting technical advances, their broader deployment in discovery workflows remains an active frontier.

##### Kinetic parameter extraction from biosensor readouts.

Nevertheless, the readouts these systems generate, whether from purely microbial or hybrid platforms, can be transformed into quantitative recovery curves that describe how regulatory signals change over time. This enables estimation of recovery rates, half-lives, and other kinetic parameters that characterize microbiome resilience. In addition to detecting inflammation, multiple circuits have been developed to detect short-chain fatty acid (SCFA) fluctuations and convert them into a graded circuit output [100]. Since SCFA levels decline during inflammation and dysbiosis, this readout can be used following perturbation to develop time courses for community recovery.

By quantifying the spatial and temporal dynamics of regulatory signals, kinetic biosensing provides a quantitative framework for comparing community stability and recovery potential across conditions, revealing underlying regulatory feedback loops, identifying key metabolic pathways that drive ecosystem recovery, and uncovering mechanisms that stabilize host–microbiome interactions. These kinetic parameters can provide direct inputs for community resilience terms and temporal constraints in computational models.

#### 3.1.4. Nanoprobe-based physical and mechanical measurements

Current gut microbiome research has largely focused on genetic composition [124], metabolic pathways [125], and chemical signaling networks [126]. Studies on bile acids, short-chain fatty acids, and other metabolites have provided important insights [127–130]. However, these approaches often treat the gut as a relatively homogeneous and static environment, overlooking its dynamic physical context [131,132]. In reality, the intestine is highly dynamic. It is shaped by continuous peristalsis, fluid flow, and pronounced spatial heterogeneity [133]. Microorganisms occupy distinct niches within the mucus layer and lumen, where they experience different levels of shear stress, viscosity, and hydrodynamic forces [132]. As has been displayed by advanced *in vitro* systems, these mechanical factors strongly influence colonization, competition, and community stability [134–136]. However, they remain difficult to probe in situ with sufficient spatial and temporal resolution.

Ingestible nanoprobes provide a system-level measurement strategy to address this gap. They enable real-time, spatially resolved interrogation of the gut’s physical microenvironment [137]. By capturing mechanical dynamics directly, these tools allow integration of physical parameters into quantitative models of microbial ecology, bridging molecular observations with a more complete systems-level understanding.

#### 3.1.5. Ingestible nanodevices categorized by sensing parameters

##### pH.

Huang et al. developed ~100 nm gold triangular nanoparticles loaded with polyaniline (PANI) as a photoacoustic probe for *in vivo* gastrointestinal pH imaging [138]. Gold provides strong photoacoustic contrast, while PANI introduces pH-dependent optical absorption. Together, these features enable quantitative signal changes across a broad physiological range (pH 1–8), with improved tissue penetration compared to conventional optical methods. Similarly, Davis et al. designed an ~80 nm silica-doped ratiometric nanosensor for precise in situ pH measurement (pH 5–8) in dynamic gastrointestinal environments. This work further demonstrates the robustness of nanoprobe-based diagnostics [139].

##### Fluid mechanics.

Sharaga et al. tracked near-infrared fluorescent SWCNTs along the intestinal lumen in real time [140]. This approach provided spatially resolved measurements of transport dynamics, residence time, and flow heterogeneity. These parameters are critical for microbial exposure and colonization stability, yet are largely inaccessible using sequencing-based methods. This highlights the unique value of nanoprobes in characterizing the gut as a dynamic physical system.

##### Viscosity.

Shan et al. used zwitterion-functionalized nanoparticles to probe intestinal mucus transport properties [141]. Their results showed that diffusion is governed more by surface hydration and interfacial interactions than by particle size alone. In this system, particle motion was translated into quantitative transport metrics. These included diffusion rate constants derived from fluorescence recovery after photobleaching (FRAP) and apparent permeability coefficients (Papp) calculated from trans-mucus flux. 1,2-dilauroyl-sn-glycero-3-phosphocholine (DLPC) nanoparticles exhibited a 6.3-fold higher Papp, a lower fluorescence decay rate (0.94 s^−1^ vs 1.26 s^−1^), and significantly reduced mucus entrapment (3.4% vs 33.6%). By decoupling mucus penetration from epithelial uptake, this study highlights mucus permeability and barrier heterogeneity as key determinants of oral transport. It also positions nanoparticles as both delivery systems and quantitative probes of mucosal physics *in vivo*.

##### How do these nanoprobes advance the discovery of gut ecosystem dynamics?.

Nanoprobe-enabled measurements move gut microbiome research beyond descriptive correlations. They enable causal analysis of how physical habitat dynamics influence microbial behavior. By temporally aligning changes in viscosity, fluid flow, and pH with shifts in microbial composition and function, nanoprobes can help determine whether environmental perturbations precede community reorganization or arise as a consequence [137]. This capability allows physical variables to be treated as independent system inputs rather than background conditions. It also provides a framework to study microbial homeostasis and resilience. For example, recovery kinetics of flow patterns and mucus rheology after perturbations—such as antibiotics or dietary changes—can be directly compared with microbial reassembly dynamics.

In this context, nanoprobes complement genetic and biochemical tools, including phage, plasmid, and biosensor systems. Rather than inferring environmental constraints indirectly, they directly quantify the physical habitat that shapes microbial behavior. This supports the development of an integrated gene–signal–physics framework for systems-level understanding. Physical parameters provided by nanoprobe measurements such as site-specific pH, fluid transit, diffusion rates, and permeability coefficients are necessary for effective parameterization of gut-on-a-chip systems and downstream predictive models in translational research. Values for these terms are often derived from the chip itself or from the literature, both of which may inadequately reflect *in vivo* conditions specific to the therapeutic application at hand. Nanoprobe studies can serve as direct validation and calibration layers for such models, grounding physical assumptions in observed gut physiology rather than *in vitro* approximations. Such calibration can improve the fidelity of predictions related to influential variables such as eLBP transit, exposure, and clearance.

### 3.2. Investigating microbial metabolic dynamics and downstream host impact

#### 3.2.1. Circuit-controlled metabolic flux

Inducible synthetic gene circuits can be used to control the production of a single metabolite, transitioning from an “on/off” mode to a system with activity levels calibrated by host physiologic signals. This is achieved by placing a metabolic pathway under an inducible ‘disease-sensing’ promoter to generate graded metabolite production rates *in vivo*.

##### Dysbiosis-triggered circuits as quantitative probes.

Inducible sensing and graded response architectures can serve a different role than the diagnostic and therapeutic applications discussed in section 2.2.2: as quantitative probes that reveal previously inaccessible dose-response relationships between microbial metabolic activity and host outcomes. One such system with potential in this regard is Koh et al.’s engineered dysbiosis-triggered *EcN* strain that modulates bile acid metabolism in response to signals of dysbiosis to mitigate *C. difficile* infection (CDI) [142]. The integrated circuit is triggered by elevated sialic acid as a dysbiosis marker, with glucose availability and colonic pH modulating the dose-dependent production of bile salt hydrolase activity that suppresses *C. difficile* spore germination. The resultant design demonstrates how dysbiosis severity and gut-region specific factors could be leveraged to signal optimal dosing and distribution. Future studies could couple this technology with reporter-only strains, graded antibiotic doses, and CDI insults to unravel dose-response dynamics. First, graded antibiotic exposure with a reporter-only strain could connect the metabolic signatures of varying severities of dysbiosis with pathogen permissiveness. Second, testing therapeutic efficacy of the engineered strain across that antibiotic dosing gradient could determine whether therapeutic efficacy scales with varying degrees of dysbiosis severity, sialic acid release, and bile acid dysregulation.

##### Circuit rewiring for hypothesis-driven discovery.

More broadly, this established circuit architecture can be re-wired to target different input/output functions and test separate hypotheses. Mechanistically, this biosensor could be used to map other gut metabolites linked to the overgrowth of certain pathogenic bacteria or to elucidate aspects of gut biology. This approach could be used to isolate specific metabolic functions carried out by ‘beneficial’ bacteria like *Akkermansia muciniphila* to establish dose dependence with respect to host outcomes [143,144]. For example, an engineered strain with a sialic acid biosensor coupled to a single biosynthetic pathway like propionate could disentangle two separate questions: whether the magnitude of removal of free sialic acid from the pathogen-accessible pool determines the strength of pathogen suppression, and whether the byproduct (propionate or acetate) independently contributes to that suppression.

#### 3.2.2. Tracking metabolic dependencies

##### Auxotrophic metabolic sensors for flux attribution.

Complementary to the circuit-based approaches described in section 2.2, engineered auxotrophs provide an alternative strategy for quantifying metabolic dependencies that shape community structure by making growth itself the readout. By constructing strains that lack vital biosynthetic pathways and pairing them with prototrophic or complementary partners, one can make growth rate and reporter output explicit functions of cross-fed metabolites. Auxotrophic metabolic sensors (AMS) have already been used to convert flux distributions into quantitative, growth-coupled signals [145]. For example, computation-guided auxotrophic designs in *E. coli* rewired central carbon metabolism so that biomass formation strictly depended on glyoxylate availability, generating sensor strains with glyoxylate sensitivities spanning three orders of magnitude [145]. Extending such auxotrophic sensor approaches to complex communities in combination with targeted perturbation of relevant strains can allow researchers to map which taxa are net producers versus consumers of specific metabolites. By quantifying which community members rescue or suppress auxotrophic growth, these sensors could reveal directionality in microbiota nutrient flow and expose competitive, commensal, or cooperative metabolic relationships that are otherwise invisible in bulk measurements.

##### Adapting AMS for in vivo community contexts.

However, translating AMS from defined laboratory conditions to the gut environment introduces several confounding variables. Dietary and host-derived amino acids could non-specifically rescue auxotrophic strains, and background metabolite concentrations may compress the dynamic range. Rather than treating these inherent gut factors solely as sources of noise, intrinsic factors such as O_2_, pH, nutrient availability, and host secretions could be deliberately integrated into AMS design to counteract or exploit these gradients, as mentioned in section 3.2.1. For instance, making the biosynthesis of an essential growth factor contingent on a signal that varies spatially (such as anaerobic conditions in the distal colon or acidic pH in the proximal gut) would force AMS strains to occupy defined spatial niches, effectively converting colonization gradients into mapping of physiologic dynamics [146]. Using such systems in a spatially controlled gut environment, such as a gnotobiotic host colonized with a defined consortium, would allow AMS-based experiments to move from artificial laboratory environments toward ecologically relevant community contexts. These experiments make spatial structure a tractable experimental variable rather than an uncontrolled confounder.

##### Combining auxotrophic and phage-based approaches.

When paired with phage-mediated knockouts, auxotroph experiments can be used to determine key producers and cross feeding dependencies within the community. While simple auxotroph studies identify whether a metabolite is produced, co-administering these strains along with targeted phage knockout of suspected producers of that metabolite can use auxotroph survival to map the net producers of a specific compound to a species or functional group within the community. The combination of these two independent methods is stronger evidence of metabolic dependency than can be provided by either method alone, as it can narrow down not only whether a metabolite is present but which taxon produces it. Beyond discovery efforts, identifying which species or functional groups serve as net producers of key metabolites can help determine whether a patient’s existing community can sustain an introduced probiotic that depends on those metabolites, directly informing personalized formulation decisions. Further, pairing both approaches with reporter readouts such as fluorescence and acoustic signals as well as targeted metabolomics can convert these experiments from qualitative community observations into quantitative transfer functions that can parameterize computational models.

Collectively, the engineering approaches outlined in this section represent an emerging toolkit with the potential to systematically address key knowledge gaps in gut microbial community dynamics. Phage perturbations can yield resilience parameters (recovery half-lives, tipping points), conjugative plasmids have demonstrated capacity to quantify gene transfer rates and niche occupancy across gut regions, kinetic biosensors can generate time-resolved regulatory curves linking metabolite fluctuations to circuit outputs, and nanoprobes have produced quantitative physical transport constants including diffusion rate coefficients and apparent permeability values in relevant gut environments. While prospective in the context of eLBP discovery, these emerging approaches have the potential to provide calibration targets that constrain computational modeling frameworks described in section IV.

## 4. Predictive modeling and quantitative validation: bridging the gap

**Fig. 2.**
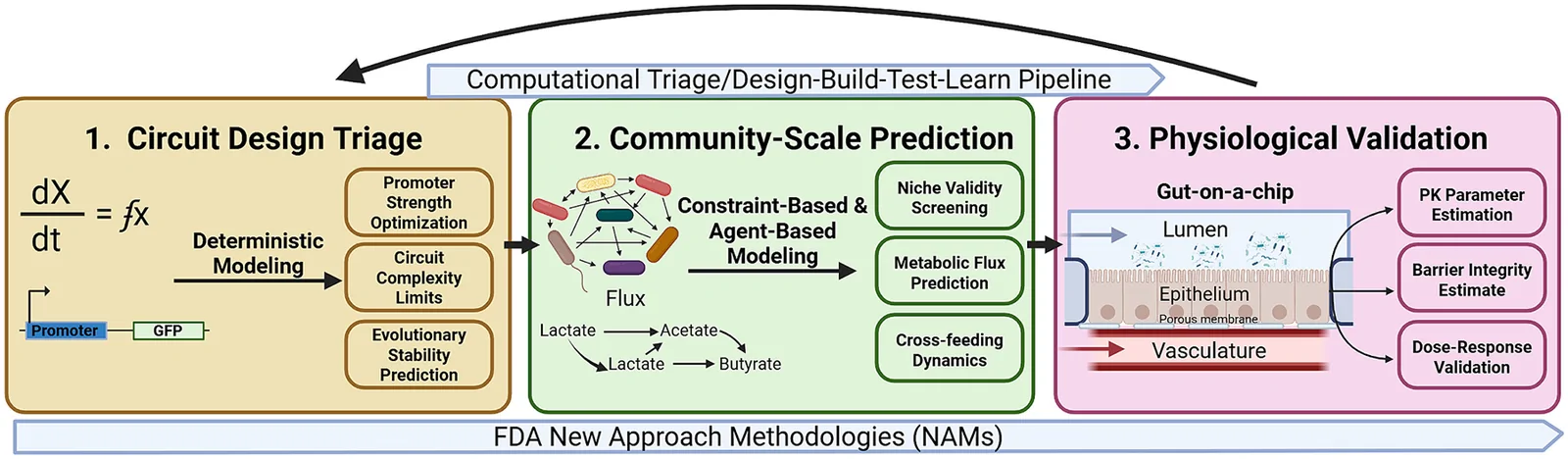
Computational triage pipeline for eLBP design within a Design-Build-Test-Learn (DBTL) framework. Stage 1: Deterministic ODE-based modeling enables a priori triage of circuit designs by informing promoter strength optimization, circuit complexity limits, and evolutionary stability. Stage 2: Constraint-based models (FBA/MICOM) predict community-scale metabolic behavior, while agent-based models generate hypotheses regarding niche validity and cross-feeding dynamics. Stage 3: Gut-on-a-chip microfluidic systems provide physiological validation through PK parameter estimation, barrier integrity assessment, and dose-response validation in a human-relevant context. This tiered approach is consistent with FDA guidance on New Approach Methodologies (NAMs) for reducing reliance on animal models in preclinical development.

While important and necessary, unraveling gut microbial dynamics through empirical physical experiments projects to be a lengthy process that delivers a narrow lens of insight. Additionally, these efforts often function on insufficient background information and context. Given the concurrent proliferation and laboratory integration of AI and ML based approaches utilizing massive training databases and computational power, deterministic and *in silico* methods can be used to accelerate and narrow down the design phase. In addition, advancements in *in vitro* modeling and biomaterials enable highly precise and targeted experiments for characterizing mechanisms of action and identifying contributing or limiting factors within the gut microenvironment. For circuit design and chassis selection, a DBTL pipeline could start by leveraging deterministic and *in silico* modeling for *a priori* triage of proposed designs and chassis compatibility. Next, advanced *in vitro* systems such as gut-on-a-chip (GOC) can assess translational potential. In line with recent FDA guidance regarding new approach methodologies (NAMs), advancement and adoption of these technologies into eLBP workflows will allow researchers to forecast translational potential early, cutting down on unnecessary resource investment into animal experiments and clinical trials unlikely to meet their endpoints [147]. These approaches also provide answers to fundamental questions which are crucial in bridging translational gaps in microbiome engineering. The proposed framework is visualized in Fig. 2.

### 4.1. Deterministic modeling of circuit performance

Prior reviews have outlined that the success of an eLBP is jointly dependent on per-cell expression kinetics, colonization rates, and physiologic complexities of the host gastrointestinal tract. This multifactorial dependency makes the translational transition the primary obstacle in eLBP design [148–151]. While empirical validation through physical experiments remains essential, researchers can mitigate some of this complexity by applying an electrical engineering framework, forecasting circuit performance and metabolic viability via ordinary differential equations (ODEs). These approaches serve as functional triage to prevent wasted efforts and resources on unviable designs and provide quantitative guidance on critical model decisions such as optimal promoter strength to avoid toxicity, determining maximum sustainable circuit complexity, and chassis selection for optimal compatibility.

Since the development of Nielsen et al’s “cello” framework for ODE based automated logic in synthetic systems in 2016 [152], permutations of ODE based approaches have become significantly more comprehensive through integration of rate modifying functions (RMFs). Recent editions have moved beyond simple transcription logic to incorporate genetic resource tradeoffs [153–155], physiologic/environmental variables [156–158], evolutionary stability [159,160], and complex multi-layered circuit logic [161]. Most recently, Sechkar et al have unified this resource aware framework into a coarse-grained model. This approach captures the dynamic interplay of ribosome competition, growth regulation, and circuit burden, which could effectively reproduce several phenomenon related to circuit-host coupling and cell growth in *E. coli* [154]. While most reports in this list are proof-of-concept or modeling studies with limited examples of application, the Cello framework produced functioning circuits in first attempts 75% of the time (45/60), with increasing internal optimization rates depending on design freedom [152,161]. Recently, this Cello framework has been integrated into a system along with large language models which translates natural language descriptions into functional designs using Cello v2.1, eliminating the need for formal languages and technical expertise [162].

To bridge the gap between gene expression and population growth-modeling, Namboothiri et al. created an environmentally aware Gene Expression Across Growth Stages (GEAGS) model that could resolve roughly 80% of batch-culture prediction failures. By integrating RMFs that account for growth-dependent environmental resource fluctuations, this model could accurately reproduce transient oscillations in circuit output, an important factor in navigating nutrient gradients of the gut environment [156]. To program for therapeutic persistence, several groups have used similar deterministic approaches to quantify population level failure trajectories [155] and design controllers that suppress cheater takeover and effectively extend therapeutic half-life by 3–5x [159].

While broad generalizability and CRISPR specific versions of these approaches are still emerging [163,164], future advancements may vastly accelerate DBTL pipelines. The application of these frameworks alongside tools such as Riglar et al’s “Represillator 2.0”, which provides quantifiable data on in vivo growth rates and niche heterogeneity, offers a path to quantifying microbial circuit output under physiologically relevant conditions. One major frontier lies in the hybridization of deterministic ODEs with stochastic simulation models, representing the next step in quantitative mechanistic discovery. This hybridization however, will likely necessitate integration of data from multiple methods of modeling, as these functions are inextricably linked to the external metabolic landscape.

### 4.2. Constraint and agent-based modeling for community wide optimization

To tailor eLBP functions and chassis selection around the complex array of interactions and metabolic crossfeeding within the gut, researchers can turn to computational metabolic models to predict eLBP behavior and screen niche validity *a priori*. With the development of the AGORA 2.0 database, genome scale metabolic models with detailed functional annotation for 1,644 named bacterial species are available for building community simulations *in silico*. Constraint based reconstruction and analysis (COBRA) based approaches, namely flux balance analysis (FBA), have been extended with multi-compartment approaches to allow for detailed stoichiometric modeling at the community level [165–168]. Further, optimized frameworks like Diener et al.’s MICOM enable estimations of steady state metabolite flux, crossfeeding, niche presence/occupancy, and metabolic output at community scale. Unlike prior systems which often end up with ‘winner take most’ dynamic where only a few fast growing bacteria grow rapidly and dominate the exchange pool, this system employs a multi-objective optimization with a cooperative tradeoff assumption that results in more realistic outcomes [167]. This tool has been successfully validated against outcome data from several human trials, in which it could predict host SCFA profiles following biotic interventions, and individualized risk of C. diff infection [169–171]. Further, newer iterations have demonstrated 75–80% accuracy in predicting probiotic engraftment, and have linked fiber-induced responses to clinically relevant biomarkers [169]. Using individualized community-level butyrate production as the representative marker, predictions were significantly correlated with several cardiometabolic outcomes in biologically plausible directions [169]. In addition to value in designing personalized biotic interventions for disease management, systematic workflows have been proposed for screening candidate probiotic therapies for mechanistic efficacy, safety, and formulation strategies [172]. Such workflows could be adapted for eLBP centric applications, such as screening chassis niche validity and biological plausibility of engineered metabolite outputs in competitive microbial communities. While these extensions require building genome-scale metabolic reconstructions with reasonable parameterization, this is an active area of methodological development. When done carefully, functional estimates can serve as priors in the integrated translational framework detailed below, and patient-specific responses can be predicted for sub-population stratification.

While these models are useful and informative, their inherent limitations and limited scope of application leave unmet needs in the current modeling toolkit. First, since solutions are generated under the steady state assumption, they cannot capture temporal dynamics or transient effects. One emerging approach which captures temporal dynamics, Brunner and Chia’s friendlyNets, accounts for temporal dynamics but has significant limitations in its comprehensiveness and computational resolution [173]. Further improvements and large scale validation will be needed before this method can be reliably applied to nuanced discovery-phase decision making. Second, FBA works under the well-mixed state assumption, meaning that estimates cannot factor in gastrointestinal morphology or cellular interactions. To model spatial dynamics, researchers currently rely on other methods, such as agent-based models (ABMs).

Agent based models simulate the collective behavior of individual bacteria and host cells by encoding rules for intracellular processes, cell-cell interactions, and cell environment responses, making them particularly suited for evaluating contact-dependent strategies such as the conjugation and phage-mediated approaches described in Section 2 [174]. Building on previous systems [175,176], models such as *Gutlogo* and *Enteroscape* incorporate increasingly realistic anatomic complexity including ileum flow and crypt-villus gradients [177,178]. These systems have been used to mirror experimental infection dynamics in model systems and generate testable predictions about spatial biofilm organization. However, these approaches rely on overly simplistic metabolic assumptions (monod kinetics) and thus lack insights into individual metabolic plasticity at the community level. While hybrid approaches represent the logical solution, the amount of complexity requires massive amounts of computational power and modular assumptions. Similar to the COMETS system, BacArena extended this approach by coupling the spatial environment on a two-dimensional grid at discrete time steps, with FBA computed for each individual agent [179,180]. Building on this foundational work, new hybrid systems like *Virtual Colon* and *MetaBiome* now couple full genome scale metabolism of individual agents with host gastrointestinal factors such as the nutrient supply, peristalsis, and mucosal dynamics [181,182]. These frameworks have generated hypotheses regarding spatial localization of host-microbe metabolic exchanges and region-specific metabolic output, providing quantitative and context specific guidance for chassis selection and niche evaluation. However, these models lack rigorous in vivo data matching, limiting their use to hypothesis generation rather than quantitative prediction. In contrast, FBA approaches like MICOM can directly constrain fluxes using measured in vivo data such as metagenomic sequencing and metabolomics, and have displayed the ability to match clinical trial outcomes. To advance confidence and utility in ABM approaches, studies retrospectively calibrating modeling parameters to match clinical trial endpoints are needed to move them from pure hypothesis generation vehicles to translational tools.

One limitation of these modeling approaches is that they often rely on simplified and generic approximations of epithelial layer and immune function within the gastrointestinal tract. Dynamic models have been developed to incorporate factors such as epithelial permeability, dietary factors, and immunity-based selection pressures, which are highly individualized and dynamic drivers of microbiome heterogeneity that will influence translational potential [183–187]. Notably, Haghebaert et al. developed a mechanistic computational framework that explicitly couples epithelial processes with microbial metabolism and luminal transport, incorporating spatially and temporally resolved variables such as nutrient flux, host metabolic responses, and immune-related signaling to reveal emergent feedback loops and dysbiotic state transitions under perturbations like low-fiber intake [188]. Complementarily, Schluter et al. proposed a theoretical model in which epithelial surfaces function as a “selectivity amplifier,” enabling hosts to favor beneficial microbes despite evolutionary pressures toward fast-growing taxa, underscoring the ecological role of host-mediated selection [189]. Collectively, these models advance microbiome engineering from purely strain-focused design and shift toward systems level, translationally grounded predictions. Furthermore, these models reinforce the need for more sophisticated *in vitro* systems.

### 4.3. Advanced microfluidic systems: gut on a chip

Gut-on-a-chip microdevices recapitulate features of the gut ecosystem with peristalsis, shear stress, and a seeded epithelial monolayer [190]. These systems are generally comprised of polymer based aligned microchannels (representing the gut lumen and blood) separated by a porous flexible membrane with computer-controlled vacuum manifolds. The epithelial lining can grow into folds that recapitulate the structure of intestinal crypt-villus structures. The resulting structures display high barrier integrity and functionally diverse epithelial cell differentiation enables them to effectively model physiologically relevant drug/nutrient metabolism as well as metabolite exchange between the gut lumen and circulation [191]. More recent iterations have expanded beyond the original monoculture design, enabling co-culture with microbial consortia, immune cells, and basolateral endothelial layers, as well as multi-compartment designs that replicate fluid exchange between the gut barrier and peripheral organs such as the liver [192–198]. These systems have been used to outline governing factors in gut function and disease, including variables which may guide therapeutic development. Such system have been applied to identify of metabolites and compounds of interest, characterize absorption kinetics, and investigate microbial interaction dynamics and gut-organ crosstalk [192,197,199–206]. Currently, these systems are frequently used by pharmaceutical companies to evaluate first pass metabolism and oral bioavailability for the prediction of pharmacokinetic profiles during drug development [207].

Gut-on-a-chip systems can simulate eLBP activity in the human body, the flux of metabolite exchange between the gut and systemic circulation, and the clearance of administered bacteria. The most pertinent example of this is when the eLBP company Synlogic used a gut on a chip platform to evaluate the efficacy of SYN5183, a simpler iteration of SYNB1618, an eLBP involved in ongoing clinical trials for the treatment of phenylketonuria (PKU) at the time [208,209]. In this study, they sought to determine whether a human gut-chip model of the small intestine could recapitulate the in vivo activity of similar eLBP designs. Using dose ranges representative of their ongoing SYNB1618 trial, they found that SYN5183 was able to achieve dose dependent depletion of phenylalanine (Phe) in gut and endothelial (blood) compartment effluents, while blunting increases in the endothelial compartment in comparison to untreated control [208]. Further, it was confirmed that the low to mid dosing range of the probiotic was cleared within 6 hours, with no detectable effects on necrosis or cell viability/stress within gut or endothelial compartments. However, with extended exposure at high doses, SYN5183 was found to impair gut barrier function and induce inflammation, but did not result in bacterial translocation.

The *in vitro* Phe consumption data were used, supplemented with literature derived kinetic values, to estimate kinetic parameters for a Michaelis Menten style predictive computational model (Vmax, km, P, D). This model was then benchmarked against a recent non-human primate (NHP) trial on SYNB1618. When used to predict a time course of blood Phe concentrations after bolus administration, this model achieved a statistically significant correlation with experimental measurements (Pearson’s *R*^2^ = 0.6950, *p*<0.0001). However, there were a few notable caveats to this finding. First, this chip system did not include coculture with other gut microbes or microbial consortia, limiting its translational relevance. Second, the gut-on-a-chip system used in these experiments was not fully anaerobic, which is presumably why the SYN5183 strain was utilized instead of SYNB1618. SYNB1618 expresses both phenylalanine ammonia lyase (PAL) and an oxygen dependent L-amino acid deaminase (LAAD), the latter of which could not be effectively parameterized in a partially aerobic environment. SYN5183, which only expresses PAL, was therefore used to model PAL pathway kinetics, meaning a literature derived value was used to predict LAAD activity rather than experimental values. In future work, microbiota co-culture could be used to validate *in silico* predictions from MICOM and deterministic models regarding niche competition and circuit performance under realistic oxygen concentrations and nutrient gradients. Nonetheless, this work serves as an important example of the capacity of modern *in vitro* systems to provide physiologically relevant functional predictions for eLBP performance within a gut ecosystem. Further, they enable systematic modulation of single environmental variables at a time, which could enable the identification of key variables in the success of cargo delivery systems such as conjugative plasmids or phages.

However, despite the model’s success at predicting outcomes in NHP trials, the subsequent Phase 3 trial ultimately failed to meet its clinical endpoint. This discrepancy illustrates the key gap motivating the framework proposed below: accurate preclinical predictions from *in vitro* data, while encouraging, have not yet translated to clinical success. To overcome this gap, we propose to leverage the entire aforementioned predictive toolkit into a pharmacological framework adapted for living therapeutics, in which preclinical parameter estimates serve as priors that are iteratively updated by clinical observations rather than treated as standalone predictions. The ad hoc parameter augmentation displayed in this case study provides an example of evidentiary integration which could be systematized into a formalized and updating framework as new data become available.

While many parts of this proposed pipeline are still premature in their development, they are becoming increasingly useful for their respective roles. Deterministic ODE models, while still far from comprehensive, can provide guidance on optimal circuit design within characterized parts libraries for *E coli* based systems. For community-based predictions, constraint-based models can screen metabolic niche validity and predict community-level metabolic outputs, while agent-based models can generate testable predictions about spatial colonization and cellular interaction dynamics. Finally, gut-on-a-chip systems provide physiologically grounded estimates of strain activity, gut residence, and net import/export of metabolites of interest to feed predictive models. Altogether, these outputs provide data-driven guidance within DBTL workflows.

Following identification of a target disease or metabolite of interest, constraint-based and agent-based modeling approaches could guide chassis selection by predicting whether a candidate strain could function and/or colonize within a simulated gut environment of the target population. Within that chassis, ODE-based circuit modeling would then inform circuit design decisions, balancing trade-offs between metabolic burden, evolutionary stability, and promoter strength. Where patient microbiome data are available, MICOM modeling could be used to confirm whether the proposed therapeutic function is biologically plausible in the patient’s specific community context. Importantly, this context could also guide patient stratification to select susceptible populations. With experimental designs that mimic dose duration and disease-state specific conditions, gut-on-a-chip systems could then be used to estimate physiological parameters. As discussed previously, these systems can yield the Vmax, Km, and permeability estimates needed to populate the initial CK/PD model described in section 5. If translation and clinical trials ensue, Bayesian updating of parameter estimates using phase 1/2 clinical data would refine parameter estimates and guide go/no-go decisions before investing in future clinical trials.

Table 3 summarizes the capabilities, validation status, and key limitations of the discussed predictive modeling approaches.

## 5. From discovery insight to translational impact

### Lessons from late-stage eLBP programs: clinical failure as a systems-engineering problem

Recent late-stage failures of individual eLBP programs have highlighted distinct gaps in predictive modeling that have inspired the conception of our framework. Here we outline the publicly available information regarding the efforts of Synlogic and Novome, which suggests that these late-stage failures reflect a pattern of promising early stage results, but insufficient performance in later phases of clinical trials. At present, the specific mechanisms underlying these failures have not been fully characterized in the public literature, but this pattern points to a fundamental gap in the field’s ability to predict patient-level outcomes from preclinical parameter estimates.

#### 5.1. Novome biotechnologies: genetic stability

Novome Biotechnologies developed an engineered *Phocaeicola vulgatus* strain to treat enteric hyperoxaluria (EH), a condition which often arises following gastric bypass surgery. The strain was designed for controlled colonization by placing essential genes under a porphyran dependent promoter, and a 5-gene oxalate degradation pathway. Preclinical rat data indicated the strain was efficacious in reducing urinary oxalate, and early phase data demonstrated that it was capable of safe, stable, reversible engraftment in healthy volunteers. While publicly available information does not describe the exact rationale for the wind down, published data indicate that issues with genetic stability and colonization in EH patients were significant concerns [210]. Phase 1 healthy volunteers showed biocontainment escape via point mutations in the porphyran sensor in 4 of 19 engrafted subjects. More substantially, samples from EH patients in Phase 2 revealed large HGT events that both disrupted NB1000S therapeutic function and disrupted the synthetic niche by transferring the porphyran polysaccharide utilization locus (PUL) to native community members. Predefined efficacy analyses showed small, statistically insignificant improvements in urinary oxalate over placebo, though post hoc analysis of engrafted patients suggested a directional signal (27% reduction, p=0.03). This unexpected HGT-mediated failure represents a modeling gap that future extensions of existing DBTL frameworks will need to address, though a comprehensive treatment of HGT modeling is beyond the scope of this review. Existing computational tools for HGT modeling are not yet predictive in nature [211], but construct design choices can meaningfully influence likelihood of HGT. Whitaker et al. demonstrated that spatially separating essential porphyran utilization genes by 200 kb reduced HGT frequency by more than 10,000-fold in vitro [210].

#### 5.2. Synlogic: cross-platform efficacy

While Synlogic Therapeutics had multiple therapeutics in its pipeline, its most clinically advanced program was SYNB1934, an engineered *EcN* strain designed to rapidly break down Phe in the gut for the management of phenylketonuria (PKU) [208,209,212,213]. As described in Section IV, the strain expressed both PAL and LAAD pathways for Phe catabolism. SYNB1934 displayed promise in not only preclinical models, but also generated sufficient evidence to proceed to pivotal phase III clinical trials. Phase 2 results successfully met the primary endpoint of D5-phenylalanine AUC reduction following a meal challenge (−42.9% for SYNB1934), directly confirming that the engineered strain was actively consuming Phe in the gut. Secondary endpoints were similarly encouraging, including a 34% mean reduction in fasting plasma Phe on an all-comers basis and a 60% response rate with −53% reduction among responders [214]. However, when continued to a multi-center, large scale phase III randomized placebo-controlled trial, it was reported that efficacy results were not likely to meet clinical endpoints and the trial was discontinued prior to completion [215]. Synlogic has since shut down operations, but their efforts have produced valuable case studies which future efforts can leverage for de-risking purposes. As previously mentioned, these translational efforts were supported by strong preclinical validation and predictive modeling with both gut-on-a-chip and NHP data. On the surface, average values in phase II were encouraging, but some important caveats remained that likely contributed to failure in phase III.

While the 60% response rate (>20% reduction in fasting plasma phe) among participants was accounted for in the responder-enrichment study design for phase III, a more fundamental challenge remained. The phase III study design required patients to remain on treatment for 3 to 15 weeks to confirm response and identify maximum tolerable dose prior to randomization to treatment or placebo for a subsequent 4-week randomized withdrawal period. In addition to introducing increased sample sizes and site variation, this was a more stringent test of colonization persistence. In Phase II, optimal treatment effects were observed during the active dose titration period of the 28-day open-label Phase 2 study. The fact that treatment effects did not persist under extended periods raises questions about the ecological compatibility of engineered EcN with the competitive human gut environment, suggesting that colonization persistence, rather than enzymatic efficacy, may have been the limiting factor. Moving forward, future eLBP trial designs should incorporate colonization kinetics monitoring as a primary covariate or stratification variable rather than an implicit assumption of delivery. A CK/PD framework that explicitly links colonization density trajectories to pharmacodynamic outcomes would provide the quantitative basis for such decisions, enabling go/no-go assessments that distinguish failures of colonization persistence from failures of therapeutic activity. The wide range of fasting Phe reductions observed in phase II SYNB1934 responders (−29% to −80%) further illustrates the importance of patient specific colonization dynamics as a likely source of variation, highlighting the need for predictive frameworks which consider these differences.

#### 5.3. A framework to model key variables

Motivated by these recent translational failures, there is a need to create a framework for the refinement of DBTL workflows. While these two cases represent distinct technical issues, they converge on one common underlying problem: insufficient consideration of cellular dynamics in anticipating patient outcomes. Addressing both classes of failure will require modeling approaches that can quantitatively predict colonization dynamics, competitive fitness, and genetic stability within the gut environment. Such approaches can identify unviable designs and flag high-risk clinical trajectories before significant resources are committed. Many existing reports have pointed to the fact that the traditional PK/PD framework cannot be applied to eLBPs due to the dynamic and animate nature of bacterial delivery systems. Variables mentioned in prior sections, such as bacterial growth, death, competition, evolution, as well as host specific factors, all play a role in values of interest such as Cmax (or equivalent), Tmax, and AUC. However, motivated by the fact that PK/PD frameworks have been successfully used to predict CAR-T therapy kinetics and resulting effects on tumor growth [216–219], Renardy et al. developed a cellular kinetics/pharmacodynamics (CK/PD) predictive modelling framework to retrospectively model butyrate production in response to VE303, a therapeutic consortium for recurrent *C. diff* infection [220]. This ODE-based model captures growth, competition, epithelial binding/unbinding, and vancomycin pretreatment effects using a combination of literature derived values (gut volume, carrying capacity, diffusion coefficients, and vancomycin clearance) and values which were derived by fitting to VE303 clinical trial data (bacterial adherence/luminal concentrations, vancomycin potency, butyrate flux, gut transit). The resultant model generated data-driven hypotheses surrounding optimization of dosing schedule to best leverage the consortia for butyrate production. A similar modeling approach was deployed by Charbonneau et al. (Synlogic), in which they developed an analogous ODE-based mechanistic model to estimate *in vivo* outcomes. Using Michaelis-Menten kinetic data derived from *in vitro* experiments, they showed that resultant ODE-based models can predict eLBP pharmacological outcomes in NHP and human trials [221].

Since this model is retrospective in nature, we propose a framework where analogous calibration approaches with the tools described in section IV can be used to populate these values either prospectively or with a Bayesian updating workflow (Table 5). While Table 4 outlines utility of readily applicable tools, Table 5 maps the full parameter space of the Renardy et al. framework to both best currently available tools and emerging approaches. Admittedly, the complete prospective pipeline remains in development and will likely have limited accuracy in near-term iterations. Nevertheless, even partial implementation could refine clinical trial approaches and flag high-risk trajectories, saving considerable resources on failed later-phase trials. Given their established predictive capacity for *in vivo* translation, *in silico* tools such as MICOM and higher resolution GOC models could be incorporated into Bayesian updating workflows with input data from phase I/II, identifying where strategic design pivots or go/no-go decisions may be warranted [170,171]. One plausible interpretation of the Synlogic phase III trial was that tradeoffs associated with the non-colonizing chassis deployed (*EcN*) resulted in insufficient luminal density/residence time, and thus therapeutic potency. While decision making at this scale involves many factors beyond model parameters, this framework can aid in data-driven go/no-go decisions by flagging potential bottlenecks such as this one early in the process.

#### 5.4. Mechanism/strategy optimization and definition of CK/PD parameters

**Fig. 3.**
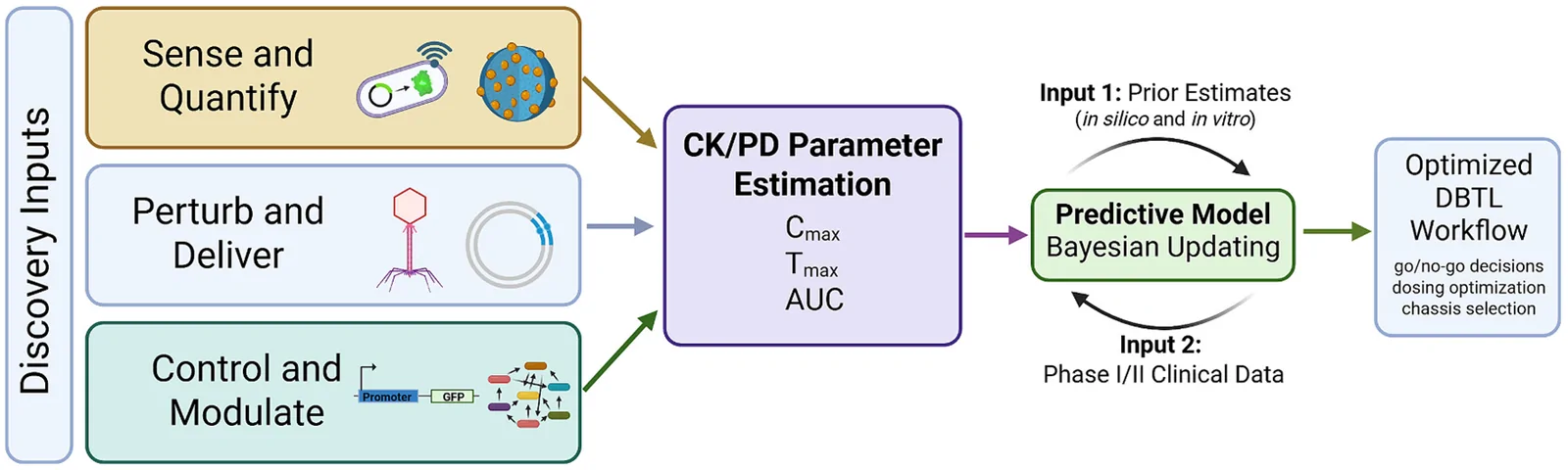
Cellular kinetics/pharmacodynamics (CK/PD) framework bridging mechanistic discovery to translational impact. Discovery inputs are organized around three functional categories: sense and quantify, perturb and deliver, and control and modulate. These categories feed into CK/PD parameter estimation for Cmax, Tmax, and AUC. In the proposed framework, these parameters would populate a predictive model through a Bayesian updating workflow that integrates prior estimates from in silico and in vitro tools with Phase I/II clinical data in an iterative refinement cycle. Once implemented, this framework is intended to inform DBTL workflow decisions including go/no-go assessments, dosing optimization, and chassis selection.

While the framework proposed by Renardy et al. represents an important step in applying pharmacological rigor to LBPs, it does not encompass added synthetic circuit functionality. For eLBPs, where therapeutic activity depends on synthetic circuit performance in conjunction with colonization and community dynamics, additional parameters must be defined that capture the relationship between circuit function and pharmacological endpoints. Future work leveraging the tools mentioned in sections 3 and 4 and the insights they can provide in gut microbial dynamics will be critical in not only informing efficacious mechanisms of action for future therapeutic efforts, but also values of interest for defining CK/PD parameters such as Cmax, Tmax, and AUC for eLBPs. Here, we map the mechanistic readouts from the proposed experimental designs to CK/PD parameters for eLBPs, as summarized in Table 6, and visualized in Fig. 3.

#### 5.5. Perturbation and delivery or targeted interventions

For approaches aimed at strategic killing for functional manipulation of the community, phage knockout studies identify functional contributions of important individual community members to resilience and metabolic output, which can directly inform 1) appropriate target strains/species to reach desired outcome, 2) downstream functional consequences, and 3) necessary doses needed to achieve desired outcome. Paired with engineered auxotrophs, differential survival following targeted killing of specific species/strains would identify key net producers and cross-feeders of metabolites of interest. Concurrently, these studies can predict whether a chassis species or eLBP function will have access to the nutrients it needs. As a result, researchers can use these insights to design consortium approaches that overcome nutrient availability issues via elimination of competitors or provision of producers. However, modeling these types of approaches remains a gap in current methods, as current ABMs lack the resolution to capture species-specific killing dynamics and their community level consequences.

#### 5.6. Sensing and quantifying the gut environment

Once targets and mechanisms have been defined, the next challenge is characterizing the gut environment where the intervention must function. Spatial specificity insights from biosensor and conjugative plasmid experiments directly inform plasmid design and chassis selection for the environmental conditions of regions of interest. Time course studies estimate Tmax directly by quantifying the temporal relationship between colonization and regional activity. If environmental characteristics revealed by nanoprobes such as viscosity and region-specific pH determine colonization stability, then formulation strategies such as synbiotics, or factors such as time of administration in relation to feeding become another priority. Further, multivariate environmental profiling with a combination of sensor panels measuring separate signals such as SCFA, calprotectin, and osmolality can generate functional networks, establishing which sensing modality provides the earliest trigger in the condition of interest.

#### 5.7. Controlling and modulating therapeutic output

After targets have been chosen and regional conditions have been characterized, the next step would be to determine whether the engineered circuit can produce sufficient therapeutic output within defined constraints. Engineered strains combining biosensor technology with therapeutic functions, such as those described in section 2.2., can establish dose-response relationships between circuit output and environmental signal strength. Particularly when done with “sense and respond” circuits, these studies can define therapeutic windows and establish Cmax-to-colonization relationships, providing quantitative estimates for parameters such as circuit complexity and promoter strength. However, circuit complexity and promoter strength also determine AUC, as they directly influence metabolic burden and evolutionary stability. ODE-based evolutionary stability models exist to quantify these tradeoffs by predicting how circuit complexity affects competitive fitness and therapeutic duration, which are critical parameters for dosing regimen design.

As mentioned, *in silico* modeling approaches do provide value in hypothesis generation and triage for proposed mechanism of action and model parameters, but several methodological limitations must be addressed before a fully prospective workflow is feasible. We categorize these limitations as 1) near-term refinements to validated tools and 2) fundamental gaps requiring novel solutions. In the short term, fidelity of validated models like MICOM and GOC can be improved with incremental extensions of validated platforms. While MICOM has shown that it can produce clinically meaningful predictions for community steady state when properly parameterized, this is dependent on accurate patient specific baseline microbial community data. The further proliferation of NGS technology and computational resources that allows for rapid individual community profiling is needed to expand its adoption. Further, incorporation of specialized eLBP metabolic functions currently requires manual construction, and MICOM is generally limited to species level predictions. Expansion of GEM databases to allow for strain level resolution and expanded parts libraries for engineered metabolic pathways will further enhance its efficacy and predictive fidelity for these applications. In the interim, emerging tools such as MIGRENE and metaGEM can be used to construct GEMs and pan models from metagenomic data to encapsulate host-specific deviations from database specifications [222–224]. These advancements, along with more widespread adoption of host derived epithelial/endothelial designs, liver compartments, and more comprehensive microbial consortia in GOC modeling will further improve de-risking capabilities in translational DBTL by improving Cmax and AUC estimate fidelity.

Other methodological limitations are caused by fundamental gaps that require novel solutions for improvement. For example, MICOM is limited in that its steady state predictions cannot capture transient effects. Interventions which intentionally use non-colonizing chassis for transient rather than durable community modulation cannot be evaluated under steady state assumption. For this limitation, dynamic FBA methods similar to the COMETS method can provide ‘real time’ estimates of growth rate and metabolic output for approaches which do not achieve durable engraftment by design. For example, *Bacillus subtilis PY79* probiotics can potently produce specific enzymes, but can only provide acute enzymatic activity [225]. An approach which can model these dynamics with significant reliability is needed for such approaches.

Additionally, the proposed framework rests on the assumption that the desired therapeutic outcome is solely local production of a molecule. This assumption breaks down for eLBPs targeting complex systemic outcomes, such as bodily metabolism. For example, an eLBP designed to secrete GLP-1 receptor agonists would aim to reduce food intake, blood glucose, and body weight, which are unlikely to be effectively predicted with current methodology. As an interim strategy to provide simpler biomarker readout surrogates for complex or nonspecific designs, MICOM simulations can identify enriched metabolites when modeling the function of interest. Similar to the butyrate-anchored approach by Renardy et al, enriched metabolites or metabolite profiles can populate the model outcome values to provide a predictable, quantifiable marker [220]. Such markers could also function as proxy indicators for optimal dosing in preclinical and early clinical studies. GLP-1 agonism in the gut may result in decreased primary bile acids with compensatory increases in secondary bile acids and/or short chain fatty acids [226,227], which are more tractable and predictable surrogate readouts than weight loss or glycemic indices.

Finally, a consequential gap that cannot be modeled with current technology and will likely require integration of ABMs, ODE predictive models, FBA, and GOC validation, is the modeling of the effects of circuit expression burden on competitive fitness and evolutionary stability within the gut community. The practical consequences of this gap are illustrated by Novome’s engineered *B. vulgatus*, which despite employing a niche-compatible chassis and multi-layered porphyran biocontainment, generated biosafety escape mutants through horizontal gene transfer [210]. This remains a consequential gap that current computational tools are incapable of capturing on the community scale. More comprehensive ODE models that can be integrated into ABMs in tandem with full GEM integration will be invaluable in iterative modeling prior to expenditure of physical resources. However, whether this level of integration is computationally feasible at community scale remains uncertain.

## 6. Discussion

Our synthesis of the literature up to this point has characterized the investigational toolkit and developed a roadmap for expanding on a preexisting framework that converts eLBP translation into a quantitative and semi-predictable process through prospective or Bayesian updating methods. Mechanistic discovery-based research using existing tools used for sensing, perturbing, and controlling variables in the gut ecosystem can create avenues for quantitative readouts which directly relate to eLBP efficacy in a CK/PD framework. As the development of this framework and the associated methodology moves along, researchers can incorporate the output values into a predictive modeling framework that will provide data-driven context for complex and highly consequential decisions regarding preclinical and clinical translation attempts. However, much of the current toolkit remains limited to broad-stroke triage rather than optimization. Achieving that resolution will require research involving eLBP calibration of computational models, consideration of functional niche availability in the design phase, and regulatory advancements.

The framing of computational tools in this context is generally that they are useful in predicting the performance of intended approaches, however this utility is bidirectional. Enterically administered probiotics or fecal microbial transplants often possess a broad and poorly understood functional profile within the gut, making their performance difficult to predict, particularly within heterogeneous gut microenvironments exhibited by recipients. Dependent on chassis and selected function, eLBPs can exhibit far more potent activities in a very simplified domain. For example, a probiotic engineered to express a single highly catalytically active enzyme produces a quantifiable, mechanistically defined perturbation [225]. This known perturbation is more predictable and functions as a ‘spike-in control’, making discrepancies between *in vivo* performance and *in silico* prediction informative rather than ambiguous. As a result, the divergence can be used to calibrate model parameters or assumptions such as community interactions, niche competition, or spatial factors. In a Bayesian updating workflow, this would in theory provide a richer calibration dataset that receives not only clinical outcome data but also a mechanistically interpretable input function. While enzyme-based approaches can be particularly useful in calibration of MICOM and stoichiometric based methods, sensing and tracking technologies such as nanoprobes and biosensors can be very useful in achieving spatial resolution in ABMs. As a result, this cycle of creating more accurate predictions for the next iteration can result in a virtuous cycle of improvement in computational contributions to DBTL workflows.

Available empirical evidence suggests that designing strategies based on microbiota functional profile is likely to have more generalizable implications than strategies based on taxonomy. It has long been documented that interindividual variation in microbiota composition far outweighs the variety in metagenomic/functional profiles, particularly at the pathway level [228]. This suggests that CK/PD parameters tied to certain metabolic ‘end point’ products are more amenable to accurate prediction and will be easier to navigate translationally. Further, it implies a ‘niche first’ engineering strategy, where initial chassis selections may place more weight on niche compatibility rather than other practical details. While only a handful of genetically tractable chassis are currently available, the prediction workflows and discovery experiments referenced in sections IV and V could be leveraged to design around the intersection of conserved functional niches within a target patient population and those that can be filled with available strains. A common theme in failed clinical translation in eLBPs is ineffective *in vivo* persistence and stability, leading to limited efficacy and evolutionary containment within the gut microenvironment. Companies such as Synlogic and Novome have achieved impressive results *in vitro*, but both models failed to meet clinical endpoints. In the case of Synlogic, it was likely that suboptimal niches for *E. coli* based techniques prevented sufficient survival and presence within the gut. In contrast, Novome selected a niche-compatible commensal (*B. vulgatus*) and achieved controlled colonization, yet the therapeutic still failed to inspire confidence, with biosafety escape mutants arising through horizontal gene transfer despite multi-layered biocontainment [210]. The contrast of these modes of failure (niche availability vs evolutionary stability) illustrate that initial niche compatibility alone is necessary but not sufficient without predictive tools to anticipate downstream barriers. A prediction toolkit based on relevant patient data and computational methods like MICOM can help researchers make balanced decisions which consider both design phase considerations such as enzyme expression levels or transport systems, and niche occupancy within the gut to optimize Cmax, Tmax, and AUC values. This conceptual shift can also help explain failures related to situations where niches are progressively filled by resident bacteria. Ultimately, this represents additional uncertainty that a dynamic discovery toolkit should be enlisted to unravel. Consideration of this additional factor on the front end can prevent unexpected failures when transitioning from an *in vitro* microbiome or model animal microbiome to human conditions.

A critical question for this framework is when and under what conditions it can be realistically implemented. At present, the modeling strategies with sufficient validation in this context only apply to well-characterized metabolite-producing eLBPs, for which GOC models and MICOM can provide kinetic estimates and community-wide predictions regarding metabolic flux and likelihood of growth where engraftment is likely or desired. Fully implementing a Bayesian updating workflow will require the field to adopt the practice of measuring colonization kinetics consistently throughout clinical trials, which is not currently standardized [5]. In the short term, incremental improvements in infrastructure would directly boost the framework’s accuracy and practical use in the clinic. These improvements include expanding GEM databases to strain level resolution, standardized colonization measurement protocols, and updated regulatory guidance on adequate pharmacokinetic characterization for living therapeutics. Down the road, fundamental modeling gaps must be resolved before the framework can account for the full range of failure modes illustrated in the Synlogic and Novome examples. Specifically, improved modeling approaches to predict HGT dynamics, evolutionary stability, and circuit performance under physiologically relevant conditions are needed. Ultimately, the framework is most readily applicable to eLBPs which produce simple, measurable outputs, and improvements in methodological and regulatory processes are needed to extend its scope of utility.

With that said, the employment of BHR conjugative plasmids represents a potential workaround for this limitation. If a chassis is not compatible with a niche for an extended period of time, perhaps horizontal gene transfer can be leveraged to impart lasting effects on resident bacterial activity. Parallel efforts exist, with some groups developing conjugation based approaches which functionally alter native gut bacteria, either by delivering CRISPRi to remove harmful functions such as genotoxin production, or adding new metabolic functions with dietary selection for enrichment [43,229].

While it is very necessary to expand the toolkit of engineered chassis in the interest of expanding opportunities in niche occupancy, strategies which optimize for ease of consistent manufacturing and simplicity should also be applied situationally. For example, *Bacillus* spores are highly thermostable, resistant to harsh conditions, but also capable of potent yet transient activity in the gut following oral administration. Additionally, other groups have proposed hydrogel encapsulation for controlled delivery. Particularly at the current moment, where biocontainment methods like ‘kill-switches’ and generalizable good manufacturing practices (GMPs) for eLBPs are still in early developmental stages, the simplicity of these strategies can serve as an important bridge for low-risk treatment for conditions where acute metabolic activity is indicated [225]. Further, they bring additional regulatory advantages such as limited risk of uncontrolled growth in the host, and the requirement for repeated dosing is attractive under conventional pharmaceutical revenue models. With that said, it remains to be seen where they stack up with highly engraftable strains with regards to Cmax/therapeutic potency.

Another limiting factor common among microbiome-based research aims is the limited available body of reproducible and consistent sequencing data for informing and training predictive models. As we move toward quantitative parameters for translation of therapeutics, it will be increasingly important for researchers in the field to adhere to specific guidelines for data handling and reporting, as is outlined in other reviews and proposals [230,231]. This variation can directly influence the accuracy of predictive modeling approaches such as MICOM, which require patient data for accuracy, and can be confounded by batch effects or discrepancies in filtering parameters. This consistency will not only play a crucial role in the adoption and generalizability of prediction models in this space, but will also work toward providing sufficient training data for machine/deep learning approaches to identify mechanisms of action likely to confer health benefits to the host. This advancement could bridge a significant gap in foundational knowledge when beginning the design phase of eLBP development. However, existing datasets also provide opportunities for significant advancement via calibration with eLBP clinical trials. Trials from companies like Novome and Synlogic contain microbiome data, and outcome data that can be used to retrospectively calibrate CK/PD models against eLBP-specific trial endpoints. Additionally, experiments aimed at calibrating MICOM predictions with GOC predictions in host-microbe coculture systems will begin to bridge the predictive gap that currently exists. Nevertheless, experiments using either abridged or full versions of the proposed workflow need to be conducted to begin validating and training models with potential clinical impact.

With that said, the current methodology available in the field can support mechanistic discovery efforts that produce quantitative eLBP equivalents of PK/PD parameters (CK/PD). This conceptual shift is consistent with the recent FDA guidance supporting the use of NAMs, and the continued expectation of eLBPs to adhere to standard biologic drug pathways with added attention to engraftment, shedding, microbiome profiling, and ecological safety [232]. Additionally, these advancements can yield significant improvements in methods which contribute to Bayesian updating and fully prospective modeling approaches which can accelerate and streamline DBTL workflows for eLBP translation. Approaching eLBP research as efforts in both translation and mechanistic discovery has the potential to position eLBPs as significant contributors to the biotechnology and biopharmaceutical landscape in the near future.

## Figures and Tables

**Table 1:** Phage subsets and characteristics.

Phage	Native Bacterial Host	Key Bacterial Receptors	Characteristics	Engineering Uses	Source
λ (lambda)	*Escherichia coli* (mainly K-12 and related strains)	uses LamB, mop, and mannose permease as receptors	Life cycle: Temperate, dsDNA	Prophage-based delivery to deliver CRISPR technologies (Base Editors, dCas9, DART, etc)	[8–10,33]
T7- phages	*E. coli* and close relatives (varies by strain)	tail fibers recognize specific outer-membrane receptors/LPS	Life cycle: Obligate lytic replication in cytoplasm, dsDNA	CRISPR-Cas9/toxin cargo for strain killing; reporter phages (GFP/luciferase); large protein display; antibacterial/cancer therapeutics	[11,12,23]
M13	F+ plasmid-containing Gram-negatives (*E. coli* mainly)	F-pilus of *E. coli*	Life cycle: Non-lytic, ssDNA	Screening and development of peptides, proteins, and antibodies using surface display via phage vector or through phagemid. Bioconjugation with nanomaterials attached at the N-terminus of pVIII.	[15–17]
Pseudomonas jumbo phages (e.g., phiKZ, MIJ3, fMGyn-Pae01)	*Pseudomonas aeruginosa* (clinical/environmental strains)	Type IV pili, flagella, LPS	Life cycle: Lytic Giant genome (>200kb)dsDNA	Antibacterial Phage therapy; chassis for large cargo (enzymes, toxins, CRISPR-13a)	[21–23]
Klebsiella phages (e.g., NAR688)	*Klebsiella pneumoniae* and related spp.	Polysaccharide capsule, LPS/O-antigens	Life cycle: Temperate “telomere” phages; dsDNA	Prospective for editing antibiotic-resistant Klebsiella spp. (prophage circuits, antivirulence)	[13]

**Table 2:** Comparative overview of engineered delivery platforms for gut microbiome applications.

Feature	Engineered Phages	Conjugative Plasmids	Nanoparticle Carriers
Delivery Efficiency	High *In Vitro;* variable *in vivo* depending on host range and MOI; lytic phages achieve near-complete target clearance at MOI > 5 (8, 74)	Moderate; in vivo transconjugant diversity (19 genera across 4 phyla demonstrated); gut environment enhances transfer relative to in vitro [40]	High; tunable surface chemistry and cargo loading enable efficient delivery, although in vivo performance depends on particle composition, targeting strategy, and biological barriers [61].
Targeting Capability	High strain specificity via tail fiber-receptor interactions; programmable host range expansion via fiber engineering; limited by community composition diversity [8,10,31]	BHR plasmids: broad cross-phyla transfer; NHR plasmids: species-restricted with greater persistence; inducible systems enable conditional transfer [37,42,75,76]	High; surface functionalization enables ligand-, antibody-, or stimuli-responsive targeting and niche-specific delivery without genetic modification, although specificity may be limited by off-target uptake and biological barriers [56].
Loading Capacity	Variable by phage type: T7 ~40 kb; jumbo phages >200 kb; M13 packaging capacity influenced by ssDNA genome architecture, phagemid systems extend functional payload range [15,16,21,22]	Large (>100 kb); accommodates multi-gene payloads including CRISPR arrays, reporters, and resistance markers simultaneously [36,77]	High and versatile; capable of co-delivering nucleic acids, proteins, small molecules, and multifunctional payloads, with loading capacity tunable through particle composition, size, and surface engineering [65].

**Table 3:** Predictive toolkit capabilities and limitations.

Modeling tier	Representative tool/platform	Primary prediction target	Validation approach	Reported predictive performance	Key limitations
Deterministic ODE (circuit-level)	Cello framework	Genetic circuit input/output behavior	Comparison of designed circuit performance to experimental measurement across user-specified circuits	75% first-build correctness, improved circuit scores in up to 32/33 benchmarks (up to 7.9-fold) [152,161]	Largely limited to *E. coli* chassis and characterized parts libraries; performance degrades for circuits operating outside the parameter space of training data
Deterministic ODE (growth-coupled expression)	GEAGS	Strain gene expression/output across growth stages	Reconciliation of model predictions with batch-culture phenotypic data	Resolved ~80% of batch-culture prediction failures relative to prior models [156]	Parameterized against batch culture data; does not capture gut-relevant conditions including oxygen gradients, substrate competition, host interactions, or compartmental pH and flow variation; largely limited to E. coli
Deterministic ODE (evolutionary stability)	Byrom & Darlington controllers; Ingram & Stan stability models	Circuit persistence under selection pressure; cheater suppression dynamics	Comparison of predicted vs observed population-level circuit retention in defined environments	Extended therapeutic half-life by 3–5x; cheater suppression controllers validated in E. coli [159,160]	Currently validated in controlled laboratory conditions; in vivo gut community validation absent; generalizability to non-E. coli chassis undemonstrated
Constraint-based community modeling	MICOM/MCMM	Community level metabolite flux; probiotic engraftment	1) Comparison of predicted growth rates to metagenomic replication rates, 2) prospective comparison to clinical trial engraftment outcomes [167]	Growth rate predictions correlated with independent metagenomic measurements, 75–80% accuracy in predicting probiotic engraftment across two clinical cohorts [167,169,170]	Steady state assumption limits capture of transient or homeostatic compensation dynamics; quality dependent on underlying genome-scale model accuracy (limited to species level resolution)
Pairwise FBA + population dynamics	friendlyNets	Probiotic engraftment in individual microbiomes	AUC-ROC analysis against experimentally observed engraftment classification [173]	AUC-ROC ~0.85 at treatment timepoint in a 22 subject *B. longum* AH1206 cohort [173]	Proof-of-concept validation limited to a single small cohort; presence/absence input ignores abundance effects; species-species interaction networks may not generalize across compositional shifts; not recommended as a primary tool where MICOM or patient metagenomic data are available [173]
Agent-based modeling	BacArena, COMETS	Spatial community dynamics; emergent ecological behavior	Qualitative recapitulation of observed community phenomena (e.g., spatial stratification, cross-feeding patterns)	Primarily hypothesis-generating; few studies report quantitative outcome prediction metrics [177–180]	High computational cost; sensitivity to initialization parameters; limited prospective validation; currently appropriate for hypothesis generation and mechanistic exploration rather than quantitative translational prediction — not recommended as a standalone decision tool in eLBP DBTL workflows at current validation levels
Gut-on-a-chip (physiological)	HuMiX, Emulate Intestine-Chip	Host-microbiome interaction; barrier function; metabolite absorption	Comparison of in vitro outputs to in vivo or clinical measurements [208]	Variable concordance; reported correlations with clinical PK parameters in narrow contexts; successfully replicated phenylalanine reduction observed in vivo for SYNB1618 but did not predict Phase 3 endpoint failure [207,208]	Often lacks systemic compartments; throughput limitations; predictive validation across diverse eLBP indications remains incomplete

**Table 4:** Readily applicable modeling methods for eLBP development: inputs, outputs, and CK/PD relevance.

Modeling Tier	Key data inputs required	Primary parameter outputs	CK/PD indicator informed	Most applicable scenario	Current Readiness
Gut-on-a-chip	Strain dosing conditions, epithelial cell types, flow parameters, disease-relevant environment	Vmax, Km, permeability (Papp), bacterial clearance rates, barrier integrity metrics, toxicity thresholds	Cmax (peak enzymatic activity under physiological conditions); Tmax (transit and clearance dynamics)	Physiological parameter estimation for CK/PD model population; safety threshold identification; engineered strain characterization before in vivo studies	High for eLBP parameter estimation — directly demonstrated with SYN5183/SYNB1618, chip-derived parameters correlated with NHP outcomes (R^2^=0.695)
Constraint-based community modeling (MICOM/MCMM)	Patient metagenomic sequencing, dietary context, AGORA 2.0 database, GEM for engineered strain	Steady-state metabolite flux, niche occupancy probability, engraftment likelihood, cross-feeding coefficients	Cmax (peak colonization density and metabolite production at steady state); patient stratification for responder prediction	Chassis niche validity screening; predicting metabolic plausibility in competitive community context; identifying permissive patient subpopulations; generating community-level priors for Bayesian updating	Moderate for eLBP — well-validated for dietary interventions and unmodified probiotics; not yet validated for engineered strains with synthetic circuits; requires eLBP-specific GEM reconstruction

**Table 5:** Computational strategy for prospective or Bayesian updating estimates.

Term	Solution in Renardy et al., 2023	Proposed tools	Prior estimate source	Refinement data (Bayesian Update)
binding rate to epithelial surface (k_on)	fit to relative abundance trajectory	GOC; ABM for spatial hypothesis generation	ABM spatial predictions, GOC epithelial adhesion assays	Phase 1 relative abundance trajectory
unbinding rate from epithelial surface (k_off)	fit to relative abundance trajectory	GOC; ABM for spatial hypothesis generation	ABM spatial predictions, GOC washout/clearance dynamics	Phase 1 relative abundance trajectory
Antimicrobial/phage kill rate (k_kill)	fit to initial butyrate drop after antibiotic pretreatment	GOC; ABM for spatial hypothesis generation	GOC dose-response co-culture, phage/conjugation delivery efficiency data	Phase 1 community shifts postintervention
butyrate production rate by host bacteria (r_host)	estimated from baseline butyrate concentrations before treatment	FBA (MICOM) -> gut on a chip	MICOM flux predictions constrained by baseline patient metagenome	Phase I/II metabolite profiling
host bacterial growth rate (μ)	estimated to match observed 8-week metabolite recovery timeline	Currently limited	MICOM growth rate estimates (limited accuracy)	Phase I metabolite recovery timelines

**Table 6:** Mapping of technique outputs to CK/PD parameters

CK/PD parameter	Important factors for eLBPs	Relevant tools/insights
Cmax	Peak colonization density, peak therapeutic output, or peak target depletion	Biosensor dose-response, MICOM colonization prediction, phage knockout for target identification, GOC for clearance
Tmax	Time to therapeutic colonization, circuit activation latency, transit to site of action	Biosensor kinetic constraints, nanoprobe transit/flow data, ABM colonization dynamics
AUC	Cumulative therapeutic exposure, evolutionary stability of circuit, persistence of colonization	ODE evolutionary stability models, MICOM niche persistence, conjugative plasmid stability data

## Data Availability

This review synthesizes published literature, no new data generated.
